# Analysis of global *Aeromonas veronii* genomes provides novel information on source of infection and virulence in human gastrointestinal diseases

**DOI:** 10.1186/s12864-022-08402-1

**Published:** 2022-02-28

**Authors:** Fang Liu, Christopher Yuwono, Alfred Chin Yen Tay, Michael C. Wehrhahn, Stephen M. Riordan, Li Zhang

**Affiliations:** 1 School of Biotechnology and Biomolecular Sciences, University of New South Wales, Sydney, NSW 2052 Australia; 2 Helicobacter Research Laboratory, School of Pathology and Laboratory Medicine, Marshall Centre for Infectious Diseases Research and Training, University of Western Australia, Perth, Australia; 3 Douglass Hanly Moir Pathology, 14 Giffnock Ave, Macquarie Park, NSW 2113 Australia; 4 Gastrointestinal and Liver Unit, Prince of Wales Hospital, University of New South Wales, Sydney, Australia

**Keywords:** *Aeromonas*, *Aeromonas veronii*, Genome, Gastroenteritis, Inflammatory bowel disease

## Abstract

**Background:**

*Aeromonas veronii* is a Gram-negative rod-shaped motile bacterium that inhabits mainly freshwater environments. *A. veronii* is a pathogen of aquatic animals, causing diseases in fish. *A. veronii* is also an emerging human enteric pathogen, causing mainly gastroenteritis with various severities and also often being detected in patients with inflammatory bowel disease. Currently, limited information is available on the genomic information of *A. veronii* strains that cause human gastrointestinal diseases.

Here we sequenced, assembled and analysed 25 genomes (one complete genome and 24 draft genomes) of *A. veronii* strains isolated from patients with gastrointestinal diseases using combine sequencing technologies from Illumina and Oxford Nanopore. We also conducted comparative analysis of genomes of 168 global *A. veronii* strains isolated from different sources.

**Results:**

We found that most of the *A. veronii* strains isolated from patients with gastrointestinal diseases were closely related to each other, and the remaining were closely related to strains from other sources. Nearly 300 putative virulence factors were identified. Aerolysin, microbial collagenase and multiple hemolysins were present in all strains isolated from patients with gastrointestinal diseases. Type III Secretory System (T3SS) in *A. veronii* was in AVI-1 genomic island identified in this study, most likely acquired via horizontal transfer from other *Aeromonas* species. T3SS was significantly less present in *A. veronii* strains isolated from patients with gastrointestinal diseases as compared to strains isolated from fish and domestic animals.

**Conclusions:**

This study provides novel information on source of infection and virulence of *A. veronii* in human gastrointestinal diseases.

**Supplementary Information:**

The online version contains supplementary material available at 10.1186/s12864-022-08402-1.

## Introduction

*Aeromonas veronii* is a Gram-negative rod-shaped motile bacterium that inhabits mainly freshwater environments such as ground water, lakes and river [1]. It has also been isolated from chlorinated and untreated drinking water [2–6]. Several *Aeromonas* species including *A. veronii* are pathogens of aquatic animals, causing diseases such as skin ulceration and systemic hemorrhagic septicemia in fish, which is a great concern in aquaculture globally [7–9].

*A. veronii* and several other *Aeromonas* species also cause human diseases. The most common diseases caused by *Aeromonas* species in humans are gastroenteritis, soft-tissue infections and bacteremia [1]. *Aeromonas* species associated human gastroenteritis are mainly caused by three *Aeromonas* species including *A. veronii*, *Aeromonas caviae* and *Aeromonas hydrophila*, with *A. veronii* being the most commonly isolated species [10]. *Aeromonas* species caused human gastrointestinal infections are positively associated with increasing age [10]. *Aeromonas* species caused gastroenteritis may present with acute or chronic courses [11–15] While most patients can recover without medical treatment, those with severe symptoms and chronic infections often require hospital admission and antibiotic therapy [14]. In addition to gastroenteritis, *Aeromonas* species were often detected in patients with inflammatory bowel disease [16].

Several studies have examined the genomes of *A. veronii* strains isolated from dairy cattle, fish, and environmental samples [17, 18]. However, limited genomic data from *A. veronii* strains isolated from patients with gastrointestinal diseases are available. In order to better understand the pathogenicity of *A. veronii* in human diseases, there is a need to examine the genomes of *A. veronii* strains isolated from patients with gastrointestinal diseases.

In this study, we sequenced, assembled and analysed 25 genomes of *A. veronii* strains isolated from fecal samples of patients with gastrointestinal diseases, including one complete and 24 draft genomes. These 25 *A. veronii* strains were identified in our previous study based on the sequences of seven housekeeping genes including *gyr*B, *rpo*D, *gyr*A, *rec*A, *dna*J, *dna*X and *atp*D [10]. Comparative genome analysis of 168 *A. veronii* strains isolated from different sources in 18 countries were also conducted.

## Results

### The complete and draft genomes of 25 *A. veronii* strains isolated from fecal samples of patients with gastroenteritis

We successfully obtained the complete genome of *A. veronii* strain A29V through hybrid assembly of the data obtained from Illumina MiSeq sequencing and Oxford Nanopore sequencing. The complete genome of *A. veronii* strain A29V had a size of 4.54 Mb, with a GC content of 58.8%. Two plasmids, designated as pAV1K and pAV7K, were identified in strain A29V, consisting of 1740 and 7073 bp respectively, with each encoding one and five proteins respectively. The pAV1K was found in another four *A. veronii* strains (pamvotica, NK02, CNRT12, and NK07), as well as other *Aeromonas* species including *Aeromonas popoffii* (strain CIP 105,493), *Aeromonas sobria* (strains 2014–10,509-27–20 and PAQ091014-19), and *Aeromonas allosaccharophila* (strain Z9-6), while pAV7K was only found in one additional *A. veronii* strain UDRT09. No potential virulence factors were identified in these two plasmids.

The detailed information of the 25 *A. veronii* genomes sequenced in this study are shown in Table 1.

**Table 1:** Summary of the 25 *Aeromonas veronii* genomes sequenced and assembled in this study

Strain name	Source	Level	Size (Mb)	GC%	N50b (bp)	No. of contigs	Plasmid	Fold coverage	Accession number of genome assembly	Accession number of raw data
A20	Human feces, gastroenteritis	Draft	4.57	58.7	199,035	42		112	JAIEXX000000000	SRR15464912
A20-10	Human feces, gastroenteritis	Draft	4.51	58.6	268,460	36		198	JAIEYE000000000	SRR15464890
A20-12	Human feces, IBD	Draft	4.46	58.9	159,675	56		291	JAIEYF000000000	SRR15464889
A20-14	Human feces, gastroenteritis	Draft	4.59	58.5	106,766	76		186	JAIEYG000000000	SRR15464888
A20-17	Human feces, gastroenteritis	Draft	4.46	58.9	116,957	70		117	JAIEYH000000000	SRR15464910
A20-5	Human feces, gastroenteritis	Draft	4.5	58.8	186,941	40		159	JAIEYI000000000	SRR15464909
A20-8	Human feces, gastroenteritis	Draft	4.4	59.0	255,850	40		208	JAIEYJ000000000	SRR15464908
A21	Human feces, gastroenteritis	Draft	4.49	58.8	118,245	80		120	JAIEXY000000000	SRR15464911
A21-10	Human feces, gastroenteritis	Draft	4.42	58.6	159,089	57		175	JAIEYK000000000	SRR15464907
A21-11	Human feces, gastroenteritis	Draft	4.44	58.9	165,092	54		143	JAIEYL000000000	SRR15464906
A21-13	Human feces, gastroenteritis	Draft	4.37	58.9	119,909	55		258	JAIEYM000000000	SRR15464905
A21-14	Human feces, gastroenteritis	Draft	4.65	58.4	104,270	82		129	JAIEYN000000000	SRR15464904
A21-15	Human feces, gastroenteritis	Draft	4.51	58.8	110,908	68		251	JAIEYO000000000	SRR15464903
A21-16	Human feces, gastroenteritis	Draft	4.37	58.8	260,229	37		325	JAIEYP000000000	SRR15464902
A21-19	Human feces, gastroenteritis	Draft	4.66	58.6	189,060	37		233	JAIEYQ000000000	SRR15464901
A21-4	Human feces, gastroenteritis	Draft	4.61	58.7	115,259	76		207	JAIEYR000000000	SRR15464899
A21-5	Human feces, gastroenteritis	Draft	4.64	58.8	255,727	48		262	JAIEYS000000000	SRR15464898
A21-6	Human feces, gastroenteritis	Draft	4.49	58.5	224,448	43		310	JAIEYT000000000	SRR15464897
A21-8	Human feces, gastroenteritis	Draft	4.52	58.8	169,064	50		136	JAIEYU000000000	SRR15464896
A26	Human feces, gastroenteritis	Draft	4.52	58.8	252,545	34		106	JAIEXZ000000000	SRR15464900
A27	Human feces, gastroenteritis	Draft	4.47	58.7	159,194	57		174	JAIEYA000000000	SRR15464894
A29V	Human feces, gastroenteritis	Complete	4.54	58.8	N/A	3	2	111	CP080630-CP080632	SRR15464895
A7	Human feces, gastroenteritis	Draft	4.52	58.9	118,736	80		130	JAIEYB000000000	SRR15464893
A8	Human feces, gastroenteritis	Draft	4.42	58.8	169,095	60		143	JAIEYC000000000	SRR15464892
A9	Human feces, gastroenteritis	Draft	4.54	58.8	166,550	51		146	JAIEYD000000000	SRR15464891

The 25 *A. veronii* strains listed in Table 1 were isolated in Australia. *IBD* inflammatory bowel disease, *N/A* not applicable

### Phylogenetic analysis of global *A. veronii* genomes

**Fig. 1:**
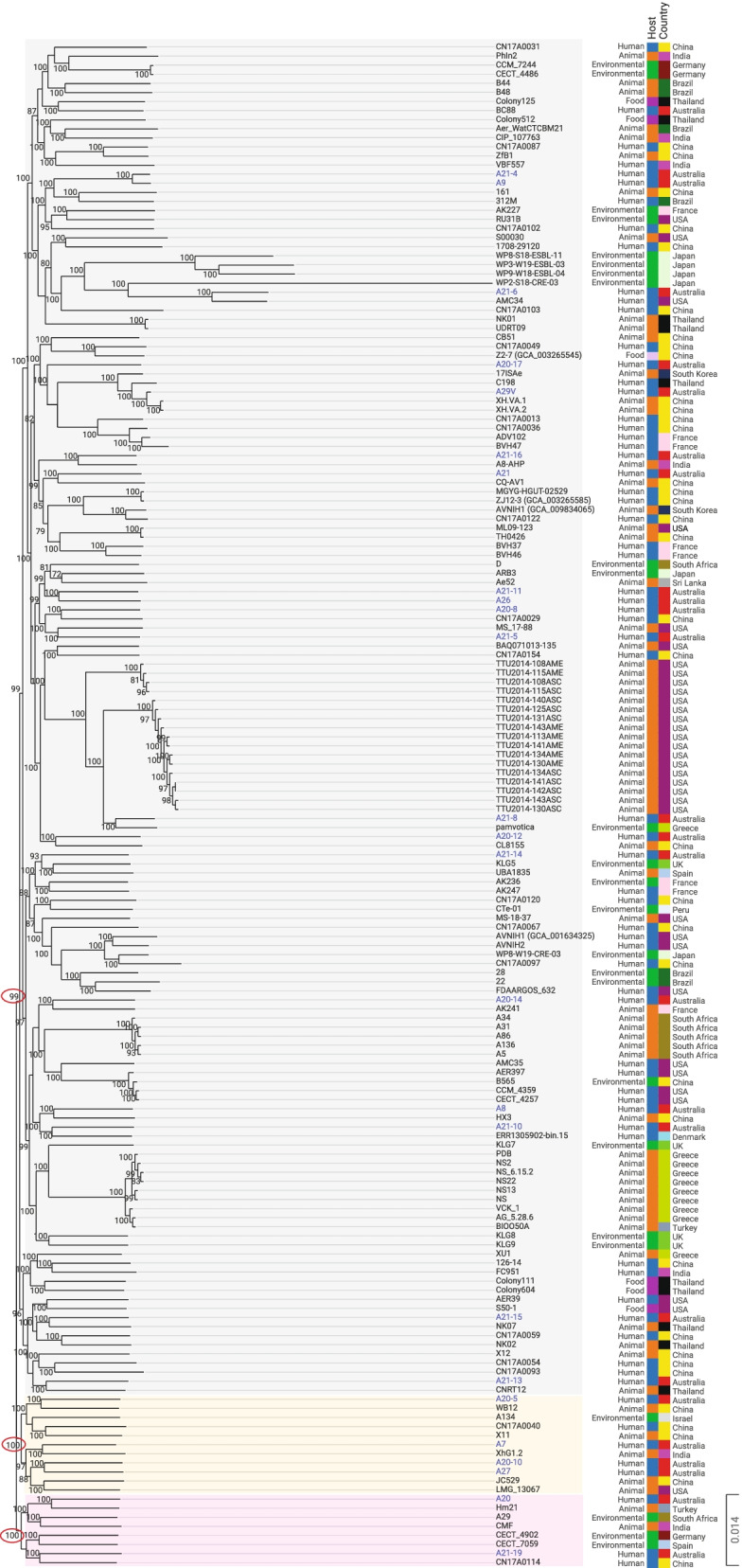
Phylogenetic tree generated based on *Aeromonas veronii* core genome. The phylogenetic tree was generated based on the core genome of 168 *A. veronii* strains isolated from different sources globally using maximum likelihood method by FastTree. The 168 *A. veronii* strains formed three clusters. Cluster 1 (shaded light grey colour, bootstrap value 99) contained 149 *A. veronii* strains, Cluster 2 (shaded yellow colour, bootstrap value 100) contained 11 strains and Cluster 3 (shaded pink colour, bootstrap value 100) contained eight strains. Within Cluster 1, strains isolated from the same environmental or animal sources often formed small groups. The genomes of *A. veronii* strains with blue colour were sequenced in this study

A total of 168 *A. veronii* genomes were used for analysis in this study, including 25 *A. veronii* genomes sequenced in this study and 143 *A. veronii* genomes obtained from public databases (Table 2). The *A. veronii* genomes in the public databases were obtained from National Center for Biotechnology Information (NCBI) genome database and their genome details and isolation sources were recorded. The core genome of the 168 *A. veronii* strains contained 1315 genes. Based on the maximum likelihood phylogenetic tree constructed from the core genome of the 168 *A. veronii* strains, three distinctive phylogenetic clusters were observed (Fig. 1). Cluster 1 contained 149 *A. veronii* strains (bootstrap value 99), which were from 18 countries. Cluster 2 (bootstrap value 100) had 11 *A. veronii* strains, which were from five countries including Australia (four strains), China (four strains), Israel (one strain), India (one strain) and USA (one strain). Cluster 3 (bootstrap value 100) contained the remaining eight *A. veronii* strains, which were from seven countries including Australia (two strains), Turkey (one strain), South Africa (one strain), India (one strain), Germany (one strain), Spain (one strain) and China (one strain). All three clusters contained strains from different sources, including humans, animals and environmental samples (Fig. 1).

**Table 2:** The 143 *Aeromonas veronii* strains in the public databases that were used in this study

Strain names	Country	Source	Level	Size (Mb)	GC %	N50 (bp)	No. of contigs	Plasmid	Ref
BC88	Australia	Human feces, dysentery	Draft	4.60	58.5	215,763	155		
FC951	India	Human feces, diarrhea	Complete	4.86	58.7	N/A	2	1	
126–14	China	Human feces, diarrhea	Draft	4.37	58.6	72,935	146		
312 M	Brazil	Human feces, gastroenteritides	Draft	4.57	58.6	502,756	14		
VBF557	India	Human feces, gastroenteritides	Draft	4.70	58.4	19,666	526		
ERR1305902-bin.15	Denmark	Human feces, diarrhea	Draft	4.11	59.4	32,267	226		
CN17A0013	China	Human feces	Draft	4.45	58.9	167,760	49		
CN17A0029	China	Human feces	Draft	4.60	58.8	2,737,631	19		
CN17A0031	China	Human feces	Draft	4.42	58.9	156,076	45		
CN17A0036	China	Human feces	Draft	4.45	58.9	230,866	38		
CN17A0040	China	Human feces	Draft	4.44	58.9	302,838	32		
CN17A0049	China	Human feces	Draft	4.30	58.9	302,677	31		
CN17A0054	China	Human feces	Draft	4.33	58.9	217,304	57		
CN17A0059	China	Human feces	Draft	4.26	58.9	164,457	48		
CN17A0067	China	Human feces	Draft	4.55	58.7	180,288	60		
CN17A0087	China	Human feces	Draft	4.58	58.6	145,832	80		
CN17A0093	China	Human feces	Draft	4.35	58.6	120,695	64		
CN17A0097	China	Human feces	Draft	4.52	58.6	141,638	88		
CN17A0102	China	Human feces	Draft	4.47	58.7	126,005	75		
CN17A0103	China	Human feces	Draft	4.43	58.8	110,083	102		
CN17A0114	China	Human feces	Draft	4.43	58.9	237,189	35		
CN17A0120	China	Human feces	Draft	4.52	58.6	82,803	128		
CN17A0122	China	Human feces	Draft	4.48	58.7	196,730	33		
CN17A0154	China	Human feces	Draft	4.44	59.0	260,206	33		
ADV102	France	Human feces	Draft	4.52	58.6	108,450	87		[19]
AMC34	USA	Human intestinal tract	Draft	4.58	58.4	219,183	1		
MGYG-HGUT-02529	China	Human gut	Draft	4.70	58.4	119,499	124		
ZJ12-3	China	Human rectal swab	Draft	4.70	58.4	119,499	124		
AVNIH1 (GCA_001634325)	USA	Human perirectal swab	Complete	4.96	58.5	N/A	2	1	[20]
AVNIH2	USA	Human perirectal swab	Draft	4.52	58.9	211,774	50		[20]
1708–29,120	China	Human cholangiolithiasis bile	Complete	4.50	58.9	N/A	1		
C198	Thailand	Human blood, septicaemia	Draft	4.58	58.6	4,550,752	3		
FDAARGOS_632	USA	Human	Complete	4.56	58.9	N/A	2	1	
CECT 4257	USA	Human sputum	Draft	4.52	58.9	148,348	52		[21]
AER39	USA	Human blood	Draft	4.42	58.8	188,051	4		[21]
AER397	USA	Human blood	Draft	4.50	58.8	645,709	5		[21]
BVH37	France	Human blood	Draft	4.46	58.8	115,181	55		[19]
BVH46	France	Human blood	Draft	4.51	58.8	215,038	39		[22]
BVH47	France	Human blood	Draft	4.64	58.9	96,732	108		[19]
AK247	France	Human forehead abscess	Draft	4.55	58.8	260,691	36		[19]
AMC35	USA	Human wound	Draft	4.57	58.5	351,392	2		[21]
CCM 4359	USA	Human sputum, drowning	Draft	4.51	58.9	245,067	56		
TTU2014-108AME	USA	Dairy cattle feces	Draft	4.53	58.7	162,342	62		[17]
TTU2014-108ASC	USA	Dairy cattle feces	Draft	4.53	58.7	187,473	58		[17]
TTU2014-113AME	USA	Dairy cattle feces	Draft	4.66	58.6	74,547	122		[17]
TTU2014-115AME	USA	Dairy cattle feces	Draft	4.53	58.7	205,013	53		[17]
TTU2014-115ASC	USA	Dairy cattle feces	Draft	4.53	58.7	233,487	52		[17]
TTU2014-125ASC	USA	Dairy cattle feces	Draft	4.68	58.6	168,256	58		[17]
TTU2014-130AME	USA	Dairy cattle feces	Draft	4.68	58.6	189,668	64		[17]
TTU2014-130ASC	USA	Dairy cattle feces	Draft	4.68	58.6	247,513	49		[17]
TTU2014-131ASC	USA	Dairy cattle feces	Draft	4.68	58.6	187,444	70		[17]
TTU2014-134AME	USA	Dairy cattle feces	Draft	4.68	58.6	204,478	50		[17]
TTU2014-134ASC	USA	Dairy cattle feces	Draft	4.68	58.6	193,661	59		[17]
TTU2014-140ASC	USA	Dairy cattle feces	Draft	4.68	58.6	148,012	81		[17]
TTU2014-141AME	USA	Dairy cattle feces	Draft	4.68	58.6	223,907	48		[17]
TTU2014-141ASC	USA	Dairy cattle feces	Draft	4.68	58.6	241,272	45		[17]
TTU2014-142ASC	USA	Dairy cattle feces	Draft	4.68	58.6	247,560	45		[17]
TTU2014-143AME	USA	Dairy cattle feces	Draft	4.68	58.6	204,478	59		[17]
TTU2014-143ASC	USA	Dairy cattle feces	Draft	4.68	58.6	202,296	54		[17]
A31	South Africa	Pig rectal swab	Draft	4.64	58.5	114,767	84		
A5	South Africa	Pig rectal swab	Draft	4.77	58.2	230,041	33		
A86	South Africa	Pig rectal swab	Draft	4.64	58.5	206,004	42		
A34	South Africa	Pig rectal swab	Draft	4.64	58.5	139,859	82		
A136	South Africa	Pig rectal swab	Draft	4.67	58.4	213,730	41		
Ae52	Sri Lanka	Carassius auratus	Draft	4.56	58.7	158,595	80		
CL8155	China	Carp gut, healthy	Draft	4.68	58.6	284,020	50		
JC529	China	Carp sepsis	Complete	4.83	58.3	N/A	1		
MS 17–88	USA	Catfish	Draft	5.18	58.2	1,334,815	13		
MS-18–37	USA	Catfish	Complete	4.68	58.6	N/A	1		
ML09-123	USA	Catfish	Draft	4.75	58.4	299,782	32		
VCK_1	Greece	*Dicentrarchus labrax* kidney, diseased	Draft	4.63	58.6	68,239	120		
PDB	Greece	*Dicentrarchus labrax* kidney, diseased	Draft	4.72	58.5	72,590	141		
AG_5.28.6	Greece	*Dicentrarchus labrax* kidney, diseased	Draft	4.61	58.6	85,872	98		
NS	Greece	*Dicentrarchus labrax* kidney, diseased	Draft	4.71	58.5	67,042	140		
NS2	Greece	*Dicentrarchus labrax* kidney, diseased	Draft	4.72	58.5	69,902	143		
NS_6.15.2	Greece	*Dicentrarchus labrax* kidney, diseased	Draft	4.72	58.5	66,300	149		
NS22	Greece	*Dicentrarchus labrax* kidney, diseased	Draft	4.74	58.4	61,224	172		
NS13	Greece	*Dicentrarchus labrax* kidney, diseased	Draft	4.67	58.6	72,418	139		
BIOO50A	Turkey	*Dicentrarchus labrax* kidney, diseased	Draft	4.61	58.6	73,700	109		
17ISAe	South Korea	Discus spleen	Complete	4.66	58.5	N/A	2	1	
A8-AHP	India	*Labeo rohita*, diseased	Complete	4.77	58.4	N/A	4	3	
UBA1835	Spain	*Anguilla anguilla* epidermal mucus	Draft	4.11	59.0	17,609	323		
ZfB1	China	Fish	Complete	4.71	58.5	N/A	1		
PhIn2	India	Fish intestinal	Draft	4.30	58.8	3789	1899		[21]
CB51	China	Grass carp	Complete	4.58	58.6	N/A	1		[17]
XH.VA.1	China	*Ictalurus punctatus*	Draft	5.36	56.5	259,638	62		
XH.VA.2	China	*Ictalurus punctatus*	Draft	4.91	58.1	259,509	48		
X11	China	*Megalobrama amblycephala*	Complete	4.28	58.8	N/A	1		
X12	China	*Megalobrama amblycephala*	Complete	4.77	58.3	N/A	1		
Aer_WatCTCBM21	Brazil	*Oreochromis niloticus*	Draft	4.60	58.7	317,324	45		
CNRT12	Thailand	*Oreochromis sp.*	Draft	4.90	58.1	265,081	479		
NK01	Thailand	*Oreochromis sp.*	Draft	4.56	58.5	171,547	95		
NK02	Thailand	*Oreochromis sp.*	Draft	4.80	58.2	110,255	400		
NK07	Thailand	*Oreochromis sp.*	Draft	4.78	58.6	214,996	46		
UDRT09	Thailand	*Oreochromis sp.*	Draft	4.61	58.5	169,295	186		
BAQ071013-135	USA	Perch head kidney	Draft	4.62	58.9	167,400	50		
B44	Brazil	*Pseudoplatystoma corruscans* kidney	Draft	4.61	58.6	290,712	51		
B48	Brazil	*Pseudoplatystoma corruscans* kidney	Draft	4.73	58.7	284,404	49		
WB12	China	Carassius auratus intestine, sick	Draft	4.52	58.8	282,522	40		
AVNIH1 (GCA_009834065)	South Korea	*Silurus asotus*	Complete	4.81	58.5	N/A	1		
TH0426	China	*Tachysurus fulvidraco*	Complete	4.92	58.3	N/A	1		
XU1	Greece	*Xiphophorus helleri* kidney	Draft	4.80	58.0	206,195	92		
XhG1.2	India	*Xiphophorus hellerii*	Draft	4.57	58.7	305,294	34		
HX3	China	Alligator	Complete	4.76	58.5	N/A	2	1	
CQ-AV1	China	*Andrias davidianus* liver	Draft	4.78	58.5	204,972	36		
161	China	*Channa argus*	Draft	4.51	58.7	312,206	28		
LMG 13,067	USA	Frog	Draft	4.74	58.4	91,946	72		[21]
S00030	USA	*Heterelmis comalensis*	Draft	4.51	58.7	237,167	21		
Hm21	Turkey	*Hirudo verbena* digestive tract	Complete	4.77	58.7	N/A	2	1	[21]
CMF	India	Insect gut	Draft	4.56	58.7	40,276	200		
CIP 107,763	India	Mosquito gut	Draft	4.43	58.8	188,049	64		[21]
AK241	France	Snail	Draft	4.60	58.6	215,278	42		[19]
B565	China	Aquaculture pond sediment	Complete	4.55	58.7	N/A	1		[18]
22	Brazil	Combined sewer	Draft	5.09	58.3	66,851	185		
28	Brazil	Combined sewer	Draft	4.97	58.5	94,322	108		
CECT 7059	Spain	Drinking water	Draft	4.81	58.4	188,889	31		
RU31B	USA	Duckweeds	Draft	4.53	58.7	73,776	93		
CECT 4902	Germany	Environment	Draft	4.64	58.4	347,677	29		[19]
AK236	France	Lake water	Draft	4.41	58.8	412,126	26		
Colony604	Thailand	Food	Draft	4.57	57.8	7493	1		
Colony111	Thailand	Food	Draft	4.58	57.8	7510	1		
Colony512	Thailand	Food	Draft	4.60	58.5	16,905	1		
Colony125	Thailand	Food	Draft	4.58	58.0	7702	1		
pamvotica	Greece	Lake Pamvotis surface sentiment	Draft	4.92	58.1	739,151	21		
A134	Israel	Lake Kinneret microcystis bloom	Draft	4.41	58.7	50,812	151		
S50-1	USA	Organic kale	Draft	4.56	58.5	104,479	130		
CTe-01	Peru	Oxidation pond	Draft	4.68	58.6	111,555	200		
ARB3	Japan	Pond water	Draft	4.54	58.8	205,115	63		[21]
Z2-7	China	Pork	Draft	4.41	58.7	265,145	48		
KLG7	UK	River Don	Draft	4.55	58.8	139,212	104		
KLG5	UK	River Don	Draft	4.74	58.5	280,270	103		
KLG8	UK	River Don	Draft	4.59	58.6	198,583	76		
KLG9	UK	River Don	Draft	4.61	58.7	180,084	74		
CECT 4486	Germany	Surface water	Draft	4.41	58.9	90,706	66		[21]
CCM 7244	Germany	Surface water	Draft	4.42	58.9	185,495	74		[17]
A29	South Africa	Surface water	Draft	4.48	58.8	165,894	54		
AK227	France	Wastewater treatment plant	Draft	4.40	58.7	105,208	67		
WP2-S18-CRE-03	Japan	Wastewater treatment plant	Complete	4.94	58.6	N/A	4	3	
WP3-W19-ESBL-03	Japan	Wastewater treatment plant	Complete	4.98	58.7	N/A	6	4	
WP8-S18-ESBL-11	Japan	Wastewater treatment plant	Complete	4.91	58.7	N/A	4	3	
WP8-W19-CRE-03	Japan	Wastewater treatment plant	Complete	4.79	58.5	N/A	6	4	
WP9-W18-ESBL-04	Japan	Wastewater treatment plant	Complete	4.93	58.7	N/A	5	4	
D	South Africa	Water	Draft	4.43	59.0	54,053	149		

There are two different strains of which both named AVNIH1, the corresponding accession numbers are indicated in brackets. *N/A* not applicable

Within Cluster 1, *A. veronii* strains isolated from environmental samples or domestic animals from the same geographic locations often formed small groups (Fig. 1). For example, 13 of the 17 *A. veronii* strains isolated from dairy cattle were in the same group (bootstrap value 100). The five strains isolated from pig rectal swabs from South Africa (A31, A5, A86, A34 and A136) were in the same group (bootstrap value 100). Similarly, the nine strains (PDB, NS2, NS6.15.2, NS22, NS13, NS, VCK1, AG5.28.6 and BIOO50A) isolated from *Dicentrarchus labrax* fish from Greece also formed their own group (bootstrap value 100) (Fig. 1).

The average nucleotide identity (ANI) values of each *A. veronii* strain against the other 167 *A. veronii* strains were mostly over 95%. An exception was strain WP2-S18-CRE-03, which was isolated from a wastewater treatment plant in Japan. This strain had ANI values 91- 92% against the other 167 *A. veroniis* strains.

### Strains closely related to *A. veronii* strains isolated from fecal samples of patients with gastrointestinal diseases

Strains that are closed related to the 31 *A. veronii* strains isolated from patients with gastrointestinal diseases were identified based on the highest ANI values. Twenty-two (71%, 22/31) closely related *A. veronii* strains were from fecal samples of other human individuals, 19 of these 22 individuals had recorded gastrointestinal diseases. Nine closely related *A. veronii* strains (29%, 9/31) were from freshwater fish or domestic animals (cattle and pig) (Table 3). Of the 26 *A. veronii* strains isolated from patients with gastrointestinal diseases in Australia, 16 strains (61.5%, 15/26) had closely related strains from patients in Australia, four strains (15.4%, 4/26) had closely related strains isolated from intestinal tract of individuals in other countries (one patient had gastroenteritis and the clinical conditions of the remaining three individuals were not known), the remaining six *A. veronii* strains (23%) had closely related strains from various sources including freshwater fish, domestic animals, leech and surface water (Table 3).

**Table 3:** Strains that are most closely related to the 31 *Aeromonas veronii* strains isolated from patients with gastrointestinal diseases

*A. veronii* strains isolated from fecal samples of patients with gastrointestinal diseases	Most closely related strain (ANI value)
^a^A20	Hm21(97.09)
^a^A20-10	^a^A7 (96.769)
^a^A20-12	^a^A21-8 (96.74)
^a^A21-14	^c^CECT 4257 (96.7)
^a^A20-17	^a^A21-13 (96.55)
^a^A20-5	^a^A7 (96.79)
^a^A20-8	^c^CN17A0029 (96.84)
^a^A21	^a^A21-5 (96.58)
^a^A21-10	A29 (96.60)
^a^A21-11	^a^A21-5 (96.7)
^a^A21-13	^a^A21-15 (96.66)
^a^A21-14	^c^CECT 4257 (96.7)
^a^A21-15	^a^A21-13 (96.58)
^a^A21-16	A8-AHP (97.5)
^a^A21-19	A136 (96.69)
^a^A21-4	^a^A9 (99.30)
^a^A21-5	^a^A21-11 (96.7)
^a^A21-6	^c^AMC34 (97.85)
^a^A21-8	TTU2014-130AME (98.1)
^a^A26	^a^A21-11 (96.64)
^a^A27	^a^A7 (96.72)
^a^A29V	XH.VA.2 (99.48)
^a^A7	^a^A20-5 (96.81)
^a^A7	^a^A20-5 (96.81)
^a^A9	^a^A21-4 (99.25)
^a^BC88	^a^A20-10 (96.56)
^b^FC951	XU1 (96.55)
^b^121-14	XU1 (96.71)
^b^312M	161 (97.16)
^b^VBF557	^a^A21-8 (96.41)
^b^ERR1305902-bin.15	^a^A8 (96.84)

^a^*A. veronii* strains isolated from feces of patients with gastrointestinal diseases in Australia

^b^strains isolated from diarrheal feces of patients from other countries

^c^strains isolated from feces of human individuals without clear clinical information

### Secretion systems

Secretion systems in the genomes of 168 *A. veronii* strains were examined. Five types of secretion systems, including Type I Secretion System (T1SS), T2SS, T3SS, T4SS and T6SS were identified in *A. veronii* (Additional file 1).

T1SS system was found in all *A. veronii* strains except strain ERR1305902-bin.15. T2SS secretion system was found in all 168 *A. veronii* strains.

T3SS was found in 106 of the 168 *A. veronii* strains (63.1%). *A. veronii* strains isolated from freshwater fish, environmental samples, domestic animals (cattle and pigs) and other animals had T3SS positivity of 84% (32/38), 60% (15/25), 100% (22/22) and 70% (7/10) respectively. The ‘other animals’ group included *A. veronii* strains isolated from mosquito gut, insect gut, *hirudo verbena* digestive tract, grass carp, *Heterelmis comalensis*, *Xiphophorus helleri*, frog, snail, *Andrias advidianus* and alligator.

**Fig. 2:**
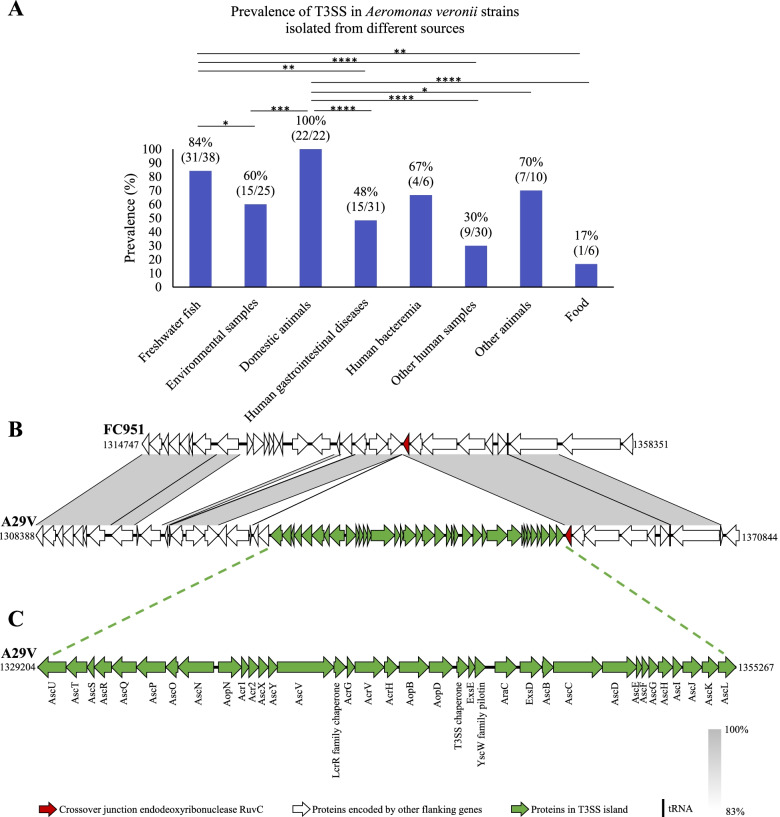
The *Aeromonas veronii* genomic island AVI-1 containing the T3SS system. The AVI-1 genomic island identified in this study contains genes encoding T3SS, which is found in 106 of the 168 strains examined in this study. **A** The prevalence of T3SS in strains isolated from patients with gastrointestinal diseases was significantly lower than that in *A. veronii* strains isolated from freshwater fish (*p* = 0.0125) and domestic animals (*p* < 0.0001). **B** Comparison of the *A. veronii* genomes with T3SS (representative strain A29V) and without T3SS (representative strain FC951) shows that the AVI-1 genomic island is located adjacent to a gene encoding crossover junction endodeoxyribonuclease (red). The identical proteins in these two strains are shaded in grey. **C** Genes in the AVI-1 genomic island that encodes T3SS components. *Indicates statistical significance (*p < 0.05; **p < 0.01; ***p < 0.001; ****p < 0.0001). Other human samples include *A. veronii* strains isolated from sputum, wound infection, cholangiolithiasis bile and fecal samples of individuals without clinical information. Other animals include *A. veronii* strains isolated from mosquito gut, insect gut, *Hirudo verbena* digestive tract, grass carp, *Heterelmis comalensis*, *Xiphophorus helleri*, frog, snail, *Andrias advidianus* and alligator. The food group included strains isolated from various food

The T3SS positivity in *A. veronii* strains isolated from patients with gastrointestinal diseases, bacteremia and other human samples was 48% (15/32), 83% (5/6) and 30% (9/30) respectively. The ‘other human sample’ group included *A. veronii* strains isolated from sputum, wound infection, bile of gallstone and fecal samples of individuals without clinical information. The T3SS positivity in *A. veronii* strains isolated from patients with gastrointestinal diseases was significantly lower than that in *A. veronii* strains isolated from freshwater fish (*p* = 0.002) and domestic animals (*p* < 0.0001). The other statistical analysis data are shown in Fig. 2A. The negativity of T3SS was confirmed by searching the franking genes in the T3SS negative strains.

A number of T4SS components were found in several *A. veronii* strains, mainly strains isolated from dairy cattle in USA. T6SS was found in 55 of the 168 *A. veronii* strains examined (32.7%) and it did not show a statistical significance in strains isolated from different sources (Additional file 1).

### T3SS in *A. veronii* is located in a genomic island that is highly similar to plasmids in *Aeromonas salmonicida*

Comparison of the genomes of 23 complete *A. veronii* genomes (11 T3SS positive and 12 T3SS negative) revealed that T3SS in *A. veronii* is located on a genomic island, which we named *A. veronii* genomic island-1 (AVI-1) (Fig. 2B). AVI-1 genomic island has a size of 26,064 bp and GC content of 60%. The AVI-1 island is adjunct to a gene encoding crossover junction endodeoxyribonuclease, an enzyme involving in homologous recombination. The components of *A. veronii* T3SS were shown in Fig. 2C.

Blast search against all bacterial genomes in public databases showed that the AVI-1 genomic island was also found in some *A. hydrophilia* and *Aeromonas salmonicida* strains. For example, the AVI-1 island is in the chromosome of *A. hydrophila* strains 23-C-23 and WCX23 (97% query coverage and 95.57% identity). In *A. salmonicida*, the AVI-1 island is in plasmids, for example plasmid pS44-3 in strain S44 and plasmid pS121-3 in strain S121 (97% query coverage and 94.85% identity).

### Virulence factors

**Fig. 3:**
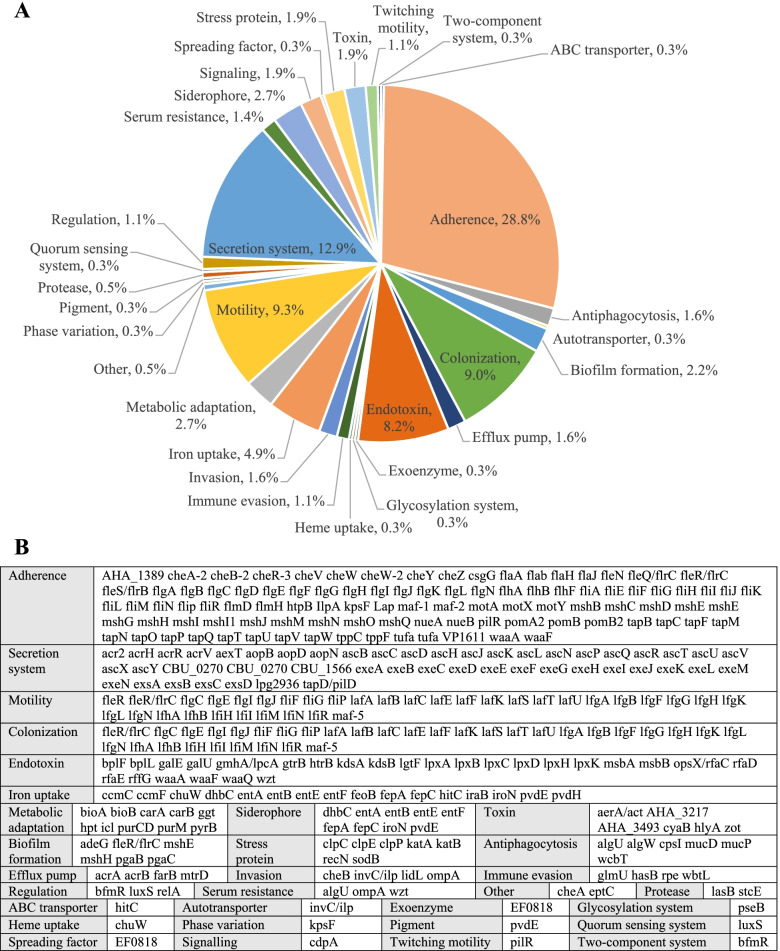
Putative virulence factors in *Aeromonas veronii* strain A29V. Putative virulence factors in the complete genome of *A. veronii* strain A29V, a strain isolated from fecal sample of a patient with gastroenteritis, was identified through searches of the Virulence Factors Database. A total of 299 putative virulence was identified. **A** Percentages of virulence factors in different categories. **B**) Virulence genes in each virulence factor category

Two hundred and ninety-nine putative virulence factors were identified in the complete genome of *A. veronii* strain A29V, including molecules involved in adherence, colonization, invasion, secretion systems, mobility, immune evasion, antiphagocytosis and others (Fig. 3).

**Fig. 4:**
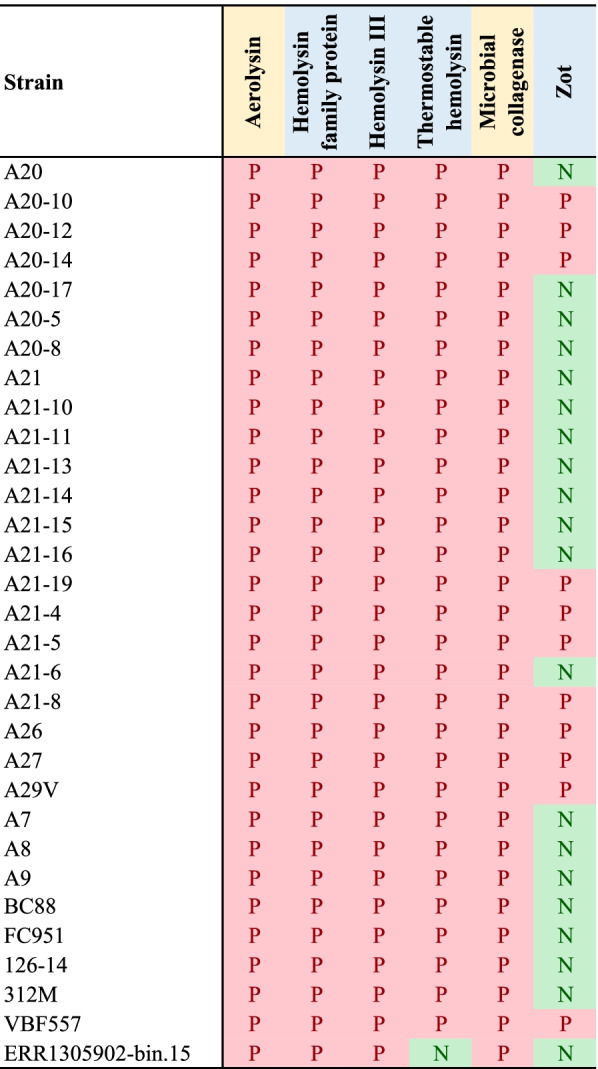
Prevalence of toxins in *Aeromonas veronii* strains isolated form fecal samples of patients with gastrointestinal diseases. Toxins identified in *A. veronii* strain A29V were further examined in other *A. veronii* strains by BLASTp. Conserved protein motifs were confirmed by pfam. Aerolysin and microbial collagenases (shaded in yellow) are secreted toxins

Toxins produced by the 31 *A. veronii* strains isolated from patients with gastrointestinal diseases were further examined. Two secreted toxins, aerolysin and microbial collagenase, were found in all 31 strains (Fig. 4). The aerolysin proteins in different *A. veronii* strains were highly similar, with the overall protein sequence identity being 75% among the 31 strains (Additional file 2). The protein sequences of aerolysin in *A. hydrophila* showed some variations, the sequence identity between *A. veronii* aerolysin and *A. hydrophila* aerolysin varied between 69 and 98%. Shiga toxin 1 (Stx1) and Shiga toxin 2 (Stx2) were not found in any of these strains. Zonula occludens toxin (Zot) was found in 11 of the 31 strains (35.5%). The Zot proteins in *A. veronii* and *Vibrio cholerae* shared 36% of protein sequence identity.

## Discussion

In this study, we sequenced and assembled 25 genomes of *A. veronii* strains isolated from fecal samples of patients with gastrointestinal infections in Australia and conducted comparative genome analysis of 168 global *A. veronii* strains, including the 25 *A. veronii* genomes that we have sequenced and additional 143 *A. veronii* strains isolated from different sources in 18 countries in Asia, Europe, Africa, Oceania, North and South America.

Twenty-five genomes, including one complete genome and 24 draft genomes of *A. veronii* strains isolated from patients with gastrointestinal diseases were successfully obtained in this study (Table 1). Despite the increasing importance of *A. veronii* in causing human gastrointestinal diseases, only six genomes of *A. veronii* strains isolated from patients with gastrointestinal diseases were available in public databases prior to this study. Our 25 *A. veronii* genomes will provide a useful source for future research on *A. veronii*.

Global *A. veronii* strains including 168 strains from 18 countries were used for phylogenetic analysis (Table 2). These 168 *A. veronii* strains formed three phylogenetic clusters based on the core genome (Fig. 1). Each cluster had *A. veronii* strains from different sources, showing the ancestors of these three clusters were not determined by the isolation sites. Most of the *A. veronii* strains (88.7%) from various sources in different countries were in Cluster 1, showing that the majority of *A. veronii* strains globally were derived from a common ancestor. Strains isolated from the same environmental or animal sources often formed small groups within Cluster 1, most likely reflecting variations in *A. veronii* isolates obtained from a single site.

The majority of the 31 *A. veronii* strains (71%) isolated from fecal samples of patients with gastrointestinal diseases were closely related to strains isolated from fecal samples of the other human individuals, most of whom had gastrointestinal diseases (Table 3). Only 29% of *A. veronii* strains isolated from fecal samples of patients with gastrointestinal diseases were closely related to strains isolated from freshwater fish and domestic animals. This interesting finding suggests that the main source for human gastrointestinal infections of *A. veronii* was not from freshwater fish or domestic animals, although they can serve as potential sources of infection. In addition to freshwater fish, domestic animals and environmental samples, *A. veronii* has also been frequently isolated from drinking water and fresh water [2–6]. Human *Aeromonas* gastrointestinal infections most often occur in warm weather [1, 10]. *Aeromonas* species and their load in different types of drinking water and fresh water that is used for preparation of food should be monitored during different seasons, which will provide further information on the main sources that cause human *Aeromonas* gastrointestinal infections.

More than half of the 168 *A. veronii* strains (63.1%) examined in this study had T3SS. T3SS is used by pathogenic bacteria to directly inject effector proteins into eukaryotic host cells, which facilitates bacterial infection of host cells or causes host cell apoptosis [23]. T3SS in *A. veronii* is located in the AVI-1 genomic island (Fig. 2). The AVI-1 genomic island is also present in the chromosome of *A. hydrophila* strains and plasmids in *A. salmonicida*, suggesting that *A. veronii* most likely has acquired T3SS via horizontal gene transfer from other *Aeromonas* species. An additional interesting finding from this study was that T3SS was significantly less present in *A. veronii* strains isolated from fecal samples of patients with gastrointestinal diseases as compared to strains isolated from freshwater fish and domestic animals (Fig. 2). This further supports the view that most of the *A. veronii* strains causing infections in human gastrointestinal tract were from a different source.

Nearly 300 putative virulence factors were identified in the complete genome of *A. veronii* strain A29V (Fig. 3). This shows that multiple virulence factors contribute to the pathogenesis of gastrointestinal diseases caused by *A. veronii*. We further examined toxins in the 31 *A. veronii* strains isolated from patients with gastrointestinal diseases. Aerolysin, a secreted toxin, is a common virulence factor presenting in all *A. veronii* strains (Fig. 4). Aerolysin is a pore-forming toxin promoting osmotic lysis of host cells. Aerolysin in *A. hydrophila* was shown to perturb human intestinal epithelial tight junction integrity and cell lesion repair [24]. The second secreted toxin, microbial collagenase, was also found in all 31 *A. veronii* strains isolated from patients with gastrointestinal diseases (Fig. 4). Bacterial collagenases degrade collagen in animal cell extracellular matrix and are important bacterial virulence factors. Microbial collagenase in *A. veronii* is involved in the pathogenesis of diseases caused by this bacterium in fish[25]. Its pathogenic role in human diseases requires further characterization. A previous study reported detection of Stx1 and Stx2 toxin genes in some human *Aeromonas* isolates [25]. However, we did not find these toxin genes in any of the 31 strains isolated from patients with gastrointestinal diseases. Zot protein was found in 35.5% *A. veronii* strains. *V. cholerae* Zot protein damages intestinal epithelial barrier tight junctions and *Campylobacter concisus* Zot protein causes intestinal epithelial cell death [26, 27]. Multiple hemolysins in *A. veronii* were identified, which were demonstrated to be virulent to host cells in other bacterial species. The levels of toxins produced by different *A. veronii* strains remain to be further examined, which may contribute to their ability in causing human gastrointestinal diseases of different severity.

## Conclusions

In summary, we report 25 genomes of *A. veronii* strains isolated from fecal samples of patients with gastrointestinal diseases, including one complete genome and 24 draft genomes. Analysis of 168 global *A. veronii* genomes including those we have sequenced show that the global *A. veronii* strains formed three clusters and the majority of *A. veronii* strains from various sources were from a common ancestor. Most of the *A. veronii* strains isolated from patients with gastrointestinal diseases were closely related to each other, with only a small percentage of these strains were closely related to *A. veronii* strains isolated from freshwater fish, domestic animals or environmental samples. Nearly 300 putative virulence factors were identified. Aerolysin, microbial collagenase and multiple hemolysins were present in all strains isolated from patients with gastrointestinal diseases. Zot toxin was only present in some strains. T3SS in *A. veronii* was in the AVI-1 genomic island identified in this study, and most likely acquired via horizontal transfer from other *Aeromonas* species and was significantly less present in *A. veronii* strains isolated from patients with gastrointestinal diseases as compared to strains isolated from freshwater fish and domestic animals. These findings provide novel information on source of infection and virulence of *A. veronii* in human gastrointestinal diseases.

## Materials and methods

### ***A. veronii*** genomes used in this study

A total of 168 *A. veronii* genomes were analysed in this study, including 25 genomes sequenced in this study and 143 genomes publicly available. Currently, there are 156 *A. veronii* genomes available in the public databases, 13 genomes were excluded from this study due to lack of information on isolation hosts or country of isolation. The 25 *A. veronii* strains sequenced in this study were isolated from fecal samples of patients with gastrointestinal diseases at the Douglass Hanly Moir Pathology laboratory in Sydney, Australia, during routine diagnostic procedure.

### Draft genome sequencing of 25 *A. veronii* strains

Sequencing and assembly of draft genomes of 25 *A. veronii* strains were conducted as described in our previous study [28]. Briefly, bacterial DNA was extracted using Gentra Puregene Yeast/Bacteria Kit (Qiagen, Chadstone, Victoria, Australia). Briefly, the DNA libraries were sequenced via the 150 bp or 250 bp paired-end sequencing chemistry on the MiSeq Personal Sequencer [29]. Reads were assembled using Shovill (v 1.0.5), and genome coverage was calculated using qualimap (v 2.2.1) [30]. Sequencing of the draft genome was performed in the Marshall Centre for Infectious Diseases Research at the University of Western Australia.

### Complete genome sequencing of *A. veronii* strain A29V

*A. veronii* strain A29V was also subjected to genome sequencing using Oxford Nanopore sequencing technique. Bacterial DNA used for this part of genome sequencing was extracted with phenol–chloroform. Libraries were prepared using the Native Barcoding Expansion kit (EXP-NBD104, Nanopore) and the Ligation Sequencing Kit (SQK-LSK109, Nanopore). The libraries were then loaded onto a R9.4 flow cell (FLO-MIN106) and sequenced on the GridION sequencing device (Nanopore). The nanopore sequencing of *A. veronii* strain A29V genome was performed at the Ramaciotti Centre for Genomics at the University of New South Wales. Basecalling were performed using Guppy (v 4.0.14). Statistics of the reads were generated using Nanostat (v 1.5.0) and genome coverage was estimated using Minimap2 (v 2.17) and qualimap (v 2.2.1) [30].

To obtain the complete genome of *A. veronii* strain A29V, the reads of *A. veronii* generated by nanopore and Illumina MiSeq were used for hybrid assembly using Unicycler (v 0.4.7). The details of hybrid assembly were described in our previous study [31].

### Annotation of the *A. veronii* genomes sequenced in this study

The complete genome of *A. veronii* strain A29V and 24 draft *A. veronii* genomes sequenced in this study were annotated using the NCBI Prokaryotic Genome Annotation Pipeline, Rapid Annotation using Subsystem Technology, and Prokka (v 1.14.5) [32–34].

### Phylogenetic analysis

Core genome was generated using Roary (v3.12.0) [35]. The maximum likelihood phylogenetic tree based on core genome was generated using FastTree (v 2.1.11) [36]. The ANI values of each *A. veronii* genome against the genomes remaining 167 *A. veronii* strains were calculated using FastANI (v 1.32) [37].

### Secretion systems

Secretion systems were examined in the genomes of 168 *A. veronii* strains. Prokka annotated protein files of the 168 *A. veronii* strains were submitted to MacSyFinder, all available protein secretion systems were searched using the default settings [38]. Visualisation of T3SS was generated using EasyFig [39]. The nucleotide sequences of *A. veronii* T3SS were searched against the genomes of all bacterial strains in NCBI non-redundant nucleotide database using BLASTn [40].

### Identification of *A. veronii* strains that were closely related to *A. veronii* strains isolated from fecal samples of patients with gastrointestinal diseases

In this study, 31 *A. veronii* strains that were isolated from fecal samples of patients with gastrointestinal diseases, including the 25 *A. veronii* strains that we have sequenced and additional six *A. veronii* strains in the public databases. The six *A. veronii* strains from public databases were strain ERR1305902-bin.15 from Denmark, strain 126–14 from China, two strains (FC951 and VBF557) from India, strain 312 M from Brazil, and a previously reported strain (BC88) from Australia.

Among the 168 *A.veronii* strains, the strain that had the highest ANI value against each of the 31 *A. veronii* strains isolated from fecal samples of patients with gastrointestinal diseases was identified as the most closely related strain.

### Putative virulence factor in *A. veronii* strains isolated from patients with gastrointestinal diseases

Putative virulence factors in the complete genome of *A. veronii* strain isolated from a patient with gastroenteritis that was sequenced in this study were firstly identified through searches of the Virulence Factors Database (VFDB) [17, 41]. The presence of toxins in the 31 *A. veronii* strains isolated from patients with gastrointestinal diseases was then searched using BLASTp, and conserved protein motifs were confirmed using pfam [40, 42].

### Statistical analysis

Fisher’s exact test (two-tailed) was used for analysis of the presence of T3SS in A*. veronii* strains isolated from different sources. *p* < 0.05 was considered to be statistically significant. Statistical analysis was performed using GraphPad Prism 7.

## Supplementary Information

**Additional file 1.**

**Additional file 2.**

## Data Availability

Genome assemblies and raw data of 25 *A. veronii* genomes sequenced in this study (one complete and 24 draft genomes) have been deposited in NCBI bacterial genome database and Sequence Read Archive database respectively. The accession numbers for the genome assemblies and raw data are available in Table 1.
