# Supplementary material for: Analysis of global Aeromonas veronii genomes provides novel information on source of infection and virulence in human gastrointestinal diseases
Source: BMC Genomics. 2022 Feb 28;23:166. doi: 10.1186/s12864-022-08402-1 (PMC8883699; doi:10.1186/s12864-022-08402-1)
Supplement: Supplementary file 1 — Additional file 1. [file 12864_2022_8402_MOESM1_ESM.pdf]

Strain	T1SS_abc	T1SS_mfp	T1SS_omf	T2SS_gspD	T2SS_gspE	T2SS_gspF	T2SS_gspG	T2SS_gspH	T2SS_gspI	T2SS_gspJ	T2SS_gspK	T2SS_gspM	T2SS_gspL	T2SS_gspN	T2SS_gspC
A20	P	P	P	P	P	P	P	P	P	P	P	P	P	P	P
A20-10	P	P	P	P	P	P	P	P	P	P	P	P	P	P	P
A20-12	P	P	P	P	P	P	P	P	P	P	P	P	P	P	P
A20-14	P	P	P	P	P	P	P	P	P	P	P	P	P	P	P
A20-17	P	P	P	P	P	P	P	P	P	P	P	P	P	P	P
A20-5	P	P	P	P	P	P	P	P	P	P	P	P	P	P	P
A20-8	P	P	P	P	P	P	P	P	P	P	P	P	P	P	P
A21	P	P	P	P	P	P	P	P	P	P	P	P	P	P	P
A21-10	P	P	P	P	P	P	P	P	P	P	P	P	P	P	P
A21-11	P	P	P	P	P	P	P	P	P	P	P	P	P	P	P
A21-13	P	P	P	P	P	P	P	P	P	P	P	P	P	P	P
A21-14	P	P	P	P	P	P	P	P	P	P	P	P	P	P	P
A21-15	P	P	P	P	P	P	P	P	P	P	P	P	P	P	P
A21-16	P	P	P	P	P	P	P	P	P	P	P	P	P	P	P
A21-19	P	P	P	P	P	P	P	P	P	P	P	P	P	P	P
A21-4	P	P	P	P	P	P	P	P	P	P	P	P	P	P	P
A21-5	P	P	P	P	P	P	P	P	P	P	P	P	P	P	P
A21-6	P	P	P	P	P	P	P	P	P	P	P	P	P	P	P
A21-8	P	P	P	P	P	P	P	P	P	P	P	P	P	P	P
A26_t	P	P	P	P	P	P	P	P	P	P	P	P	P	P	P
A27_t	P	P	P	P	P	P	P	P	P	P	P	P	P	P	P
A29V	P	P	P	P	P	P	P	P	P	P	P	P	P	P	P
A7	P	P	P	P	P	P	P	P	P	P	P	P	P	P	P
A8	P	P	P	P	P	P	P	P	P	P	P	P	P	P	P
A9	P	P	P	P	P	P	P	P	P	P	P	P	P	P	P
BC88	P	P	P	P	P	P	P	P	P	P	P	P	P	P	P
FC951	P	P	P	P	P	P	P	P	P	P	P	P	P	P	P
126-14	P	P	P	P	P	P	P	P	P	P	P	P	P	P	P
312M	P	P	P	P	P	P	P	P	P	P	P	P	P	P	P
VBF557	P	P	P	P	P	P	P	P	P	P	P	P	P	P	P
ERR1305902-bin.15	N	N	N	P	P	P	P	P	P	P	P	P	P	P	P
CN17A0013	P	P	P	P	P	P	P	P	P	P	P	P	P	P	P
CN17A0029	P	P	P	P	P	P	P	P	P	P	P	P	P	P	P
CN17A0031	P	P	P	P	P	P	P	P	P	P	P	P	P	P	P
CN17A0036	P	P	P	P	P	P	P	P	P	P	P	P	P	P	P
CN17A0040	P	P	P	P	P	P	P	P	P	P	P	P	P	P	P
CN17A0049	P	P	P	P	P	P	P	P	P	P	P	P	P	P	P

Strain	T3SS_sctC	T3SS_sctJ	T3SS_sctN	T3SS_sctQ	T3SS_sctR	T3SS_sctS	T3SS_sctT	T3SS_sctU	T3SS_sctV	T4SS_virb4	T4SS_t4cp1	T4SS_MOBB	T4SS_virB1	T4SS_virB10	T4SS_virB11	T4SS_virB2	T4SS_virB3	T4SS_virB5	T4SS_virB6	T4SS_virB8	T4SS_virB9
A20	P	P	P	P	P	P	P	P	P	N	N	N	N	N	N	N	N	N	N	N	N
A20-10	N	N	N	N	N	N	N	N	N	N	N	N	N	N	N	N	N	N	N	N	N
A20-12	P	P	P	P	P	P	P	P	P	N	N	N	N	N	N	N	N	N	N	N	N
A20-14	P	P	P	P	P	P	P	P	P	N	N	N	N	N	N	N	N	N	N	N	N
A20-17	P	P	P	P	P	P	P	P	P	P	N	N	N	P	P	P	N	P	P	P	P
A20-5	N	N	N	N	N	N	N	N	N	N	N	N	N	N	N	N	N	N	N	N	N
A20-8	N	N	N	N	N	N	N	N	N	N	N	N	N	N	N	N	N	N	N	N	N
A21	N	N	N	N	N	N	N	N	N	N	N	N	N	N	N	N	N	N	N	N	N
A21-10	N	N	N	N	N	N	N	N	N	N	N	N	N	N	N	N	N	N	N	N	N
A21-11	N	N	N	N	N	N	N	N	N	N	N	N	N	N	N	N	N	N	N	N	N
A21-13	N	P	P	P	P	P	P	P	P	N	N	N	N	N	N	N	N	N	N	N	N
A21-14	N	N	N	N	N	N	N	N	N	N	N	N	N	N	N	N	N	N	N	N	N
A21-15	P	P	P	P	P	P	P	P	P	N	N	N	N	N	N	N	N	N	N	N	N
A21-16	P	P	P	P	P	P	P	P	P	N	N	N	N	N	N	N	N	N	N	N	N
A21-19	P	P	P	P	P	P	P	P	P	N	N	N	N	N	N	N	N	N	N	N	N
A21-4	P	P	P	P	P	P	P	P	P	N	N	N	N	N	N	N	N	N	N	N	N
A21-5	N	N	N	N	N	N	N	N	N	N	N	N	N	N	N	N	N	N	N	N	N
A21-6	N	N	N	N	N	N	N	N	N	N	N	N	N	N	N	N	N	N	N	N	N
A21-8	P	P	P	P	P	P	P	P	P	N	N	N	N	N	N	N	N	N	N	N	N
A26_t	P	P	P	P	P	P	P	P	P	N	N	N	N	N	N	N	N	N	N	N	N
A27_t	N	N	N	N	N	N	N	N	N	N	N	N	N	N	N	N	N	N	N	N	N
A29V	P	P	P	P	P	P	P	P	P	N	N	N	N	N	N	N	N	N	N	N	N
A7	N	N	N	N	N	N	N	N	N	N	N	N	N	N	N	N	N	N	N	N	N
A8	N	N	N	N	N	N	N	N	N	N	N	N	N	N	N	N	N	N	N	N	N
A9	P	P	P	P	P	P	P	P	P	N	N	N	N	N	N	N	N	N	N	N	N
BC88	N	N	N	N	N	N	N	N	N	N	N	N	N	N	N	N	N	N	N	N	N
FC951	N	N	N	N	N	N	N	N	N	N	N	N	N	N	N	N	N	N	N	N	N
126-14	N	N	N	N	N	N	N	N	N	N	N	N	N	N	N	N	N	N	N	N	N
312M	P	P	P	P	P	P	P	P	P	N	N	N	N	N	N	N	N	N	N	N	N
VBF557	P	P	P	P	P	P	P	P	N	P	P	P	N	N	N	N	N	N	N	N	N
ERR1305902-bin.15	N	N	N	N	N	N	N	N	N	N	N	N	N	N	N	N	N	N	N	N	N
CN17A0013	P	P	P	P	P	P	P	P	P	N	N	N	N	N	N	N	N	N	N	N	N
CN17A0029	N	N	N	N	N	N	N	N	N	N	N	N	N	N	N	N	N	N	N	N	N
CN17A0031	N	N	N	N	N	N	N	N	N	N	N	N	N	N	N	N	N	N	N	N	N
CN17A0036	P	P	P	P	P	P	P	P	P	N	N	N	N	N	N	N	N	N	N	N	N
CN17A0040	N	N	N	N	N	N	N	N	N	N	N	N	N	N	N	N	N	N	N	N	N
CN17A0049	N	N	N	N	N	N	N	N	N	N	N	N	N	N	N	N	N	N	N	N	N

Strain	T6SSi_tssA	T6SSi_evpJ	T6SSi_tssB	T6SSi_tssC	T6SSi_tssD	T6SSi_tssE	T6SSi_tssF	T6SSi_tssG	T6SSi_tssH	T6SSi_tssI	T6SSi_tssJ	T6SSi_tssK	T6SSi_tssL	T6SSi_tssM
A20	N	N	N	N	N	N	N	N	N	N	N	N	N	N
A20-10	N	N	N	N	N	N	N	N	N	N	N	N	N	N
A20-12	P	P	P	P	P	P	P	P	P	P	P	P	P	P
A20-14	P	P	P	P	P	P	P	P	P	P	P	P	P	P
A20-17	N	N	N	N	N	N	N	N	N	N	N	N	N	N
A20-5	N	N	N	N	N	N	N	N	N	N	N	N	N	N
A20-8	N	N	N	N	N	N	N	N	N	N	N	N	N	N
A21	N	N	N	N	N	N	N	N	N	N	N	N	N	N
A21-10	N	N	N	N	N	N	N	N	N	N	N	N	N	N
A21-11	N	N	N	N	N	N	N	N	N	N	N	N	N	N
A21-13	N	N	N	N	N	N	N	N	N	N	N	N	N	N
A21-14	N	N	N	N	N	N	N	N	N	N	N	N	N	N
A21-15	N	N	N	N	N	N	N	N	N	N	N	N	N	N
A21-16	N	N	N	N	N	N	N	N	N	N	N	N	N	N
A21-19	N	N	N	N	N	N	N	N	N	N	N	N	N	N
A21-4	P	P	P	P	P	P	P	P	P	P	P	P	P	P
A21-5	N	N	N	N	N	N	N	N	N	N	N	N	N	N
A21-6	P	P	P	P	N	P	P	P	P	P	P	P	P	P
A21-8	P	P	P	P	P	P	P	P	P	P	P	P	P	P
A26_t	N	N	N	N	N	N	N	N	N	N	N	N	N	N
A27_t	N	N	N	N	N	N	N	N	N	N	N	N	N	N
A29V	N	N	N	N	N	N	N	N	N	N	N	N	N	N
A7	N	N	N	N	N	N	N	N	N	N	N	N	N	N
A8	N	N	N	N	N	N	N	N	N	N	N	N	N	N
A9	P	P	P	P	P	P	P	P	P	P	P	P	P	P
BC88	N	N	N	N	N	N	N	N	N	N	N	N	N	N
FC951	N	N	N	N	N	N	N	N	N	N	N	N	N	N
126-14	N	N	N	N	N	N	N	N	N	N	N	N	N	N
312M	P	P	P	P	P	P	P	P	P	N	P	P	P	P
VBF557	P	P	P	P	P	P	P	P	P	P	P	P	P	N
ERR1305902-bin.15	N	N	N	N	N	N	N	N	N	N	N	N	N	N
CN17A0013	N	N	N	N	N	N	N	N	N	N	N	N	N	N
CN17A0029	N	N	N	N	N	N	N	N	N	N	N	N	N	N
CN17A0031	N	N	N	N	N	N	N	N	N	N	N	N	N	N
CN17A0036	N	N	N	N	N	N	N	N	N	N	N	N	N	N
CN17A0040	N	N	N	N	N	N	N	N	N	N	N	N	N	N
CN17A0049	N	N	N	N	N	N	N	N	N	N	N	N	N	N

CN17A0054	P	P	P	P	P	P	P	P	P	P	P	P	P	P	P	N	N	N	N	N	N	N	N	N	N	N	N	N	N	N	N	N	N	N	N	N	N	N	N	N	N	N	N	N	N	N	N	N	N	N
CN17A0059	P	P	P	P	P	P	P	P	P	P	P	P	P	P	P	P	P	P	P	P	P	P	P	P	N	N	N	N	N	N	N	N	N	N	N	N	N	N	N	N	N	N	N	N	N	N	N	N	N	N
CN17A0067	P	P	P	P	P	P	P	P	P	P	P	P	P	P	P	N	N	N	N	N	N	N	N	N	N	N	N	N	N	N	N	N	N	N	N	N	N	N	N	N	N	N	N	N	N	N	N	N	N	N
CN17A0087	P	P	P	P	P	P	P	P	P	P	P	P	P	P	P	P	P	P	P	P	P	P	P	P	N	N	N	N	N	N	N	N	N	N	N	N	N	N	N	N	N	N	N	N	N	N	N	N	N	N
CN17A0093	P	P	P	P	P	P	P	P	P	P	P	P	P	P	P	N	N	N	N	N	N	N	N	N	N	N	N	N	N	N	N	N	N	N	N	N	N	N	N	N	N	N	N	N	N	N	N	N	N	N
CN17A0097	P	P	P	P	P	P	P	P	P	P	P	P	P	P	P	N	N	N	N	N	N	N	N	N	N	N	N	N	N	N	N	N	N	N	N	N	N	N	N	N	N	N	N	N	N	N	N	N	N	N
CN17A0103	P	P	P	P	P	P	P	P	P	P	P	P	P	P	P	N	N	N	N	N	N	N	N	N	N	N	N	N	N	N	N	N	N	N	N	N	N	N	N	N	N	N	N	N	N	N	N	N	N	N
CN17A0102	P	P	P	P	P	P	P	P	P	P	P	P	P	P	P	N	N	N	N	N	N	N	N	N	N	N	N	N	N	N	N	N	N	N	N	N	N	N	N	N	N	N	N	N	N	N	N	N	N	N
CN17A0114	P	P	P	P	P	P	P	P	P	P	P	P	P	P	P	P	P	P	P	P	P	P	P	P	N	N	N	N	N	N	N	N	N	N	N	N	N	N	N	N	N	N	N	N	N	N	N	N	N	N
CN17A0120	P	P	P	P	P	P	P	P	P	P	P	P	P	P	P	N	N	N	N	N	N	N	N	N	N	N	N	N	N	N	N	N	N	N	N	N	N	N	N	N	N	N	N	N	N	N	N	N	N	N
CN17A0122	P	P	P	P	P	P	P	P	P	P	P	P	P	P	P	P	P	P	P	P	P	P	P	P	N	N	N	N	N	N	N	N	N	N	N	N	N	N	N	N	N	N	N	N	N	N	N	N	N	N
CN17A0154	P	P	P	P	P	P	P	P	P	P	P	P	P	P	P	N	N	N	N	N	N	N	N	N	N	N	N	N	N	N	N	N	N	N	N	N	N	N	N	N	N	N	N	N	N	N	N	N	N	N
ADV102	P	P	P	P	P	P	P	P	P	P	P	P	P	P	P	P	P	P	P	P	P	P	P	P	N	N	N	N	N	N	N	N	N	N	N	N	N	N	N	N	N	N	N	N	N	N	N	N	N	N
AMC34	P	P	P	P	P	P	P	P	P	P	P	P	P	P	P	N	N	N	N	N	N	N	N	N	N	N	N	N	N	N	N	N	N	N	N	N	P	P	P	P	P	P	P	P	P	P	P	P	P	P
MGYG-HGUT-02529	P	P	P	P	P	P	P	P	P	P	P	P	P	P	P	N	P	P	P	P	P	P	P	P	N	N	N	N	N	N	N	N	N	N	N	N	N	N	N	N	N	N	N	N	N	N	N	N	N	N
ZJ12-3	P	P	P	P	P	P	P	P	P	P	P	P	P	P	P	N	P	P	P	P	P	P	P	P	N	N	N	N	N	N	N	N	N	N	N	N	N	N	N	N	N	N	N	N	N	N	N	N	N	N
AVNIH1 (GCA_001634325)	P	P	P	P	P	P	P	P	P	P	P	P	P	P	P	N	N	N	N	N	N	N	N	N	P	P	P	N	N	N	N	N	N	N	N	N	N	N	N	N	N	N	N	N	N	N	N	N	N	N
AVNIH2	P	P	P	P	P	P	P	P	P	P	P	P	P	P	P	N	N	N	N	N	N	N	N	N	N	N	N	N	N	N	N	N	N	N	N	N	N	N	N	N	N	N	N	N	N	N	N	N	N	N
1708-29120	P	P	P	P	P	P	P	P	P	P	P	P	P	P	P	N	N	N	N	N	N	N	N	N	N	N	N	N	N	N	N	N	N	N	N	N	N	N	N	N	N	N	N	N	N	N	N	N	N	N
C198	P	P	P	P	P	P	P	P	P	P	P	P	P	P	P	P	P	P	P	P	P	P	P	P	N	N	N	N	N	N	N	N	N	N	N	N	N	N	N	N	N	N	N	N	N	N	N	N	N	N
FDAARGOS_632	P	P	P	P	P	P	P	P	P	P	P	P	P	P	P	N	N	N	N	N	N	N	N	N	N	N	N	N	N	N	N	N	N	N	N	N	N	N	N	N	N	N	N	N	N	N	N	N	N	N
CECT 4257	P	P	P	P	P	P	P	P	P	P	P	P	P	N	P	N	N	N	N	N	N	N	N	N	N	N	N	N	N	N	N	N	N	N	N	N	N	N	N	N	N	N	N	N	N	N	N	N	N	N
AER39	P	P	P	P	P	P	P	P	P	P	P	P	P	P	P	P	P	P	P	P	P	P	P	P	N	N	N	N	N	N	N	N	N	N	N	N	N	N	N	N	N	N	N	N	N	N	N	N	N	N
AER397	P	P	P	P	P	P	P	P	P	P	P	P	P	P	P	N	N	N	N	N	N	N	N	N	N	N	N	N	N	N	N	N	N	N	N	N	N	N	N	N	N	N	N	N	N	N	N	N	N	N
BVH37	P	P	P	P	P	P	P	P	P	P	P	P	P	P	P	P	P	P	P	P	P	P	P	P	N	N	N	N	N	N	N	N	N	N	N	N	P	P	P	P	P	P	P	P	P	P	P	P	P	P
BVH46	P	P	P	P	P	P	P	P	P	P	P	P	P	P	P	N	P	P	P	P	P	P	P	P	N	N	N	N	N	N	N	N	N	N	N	N	P	P	P	P	P	P	P	P	P	P	P	P	P	P
BVH47	P	P	P	P	P	P	P	P	P	P	P	P	P	P	P	P	P	P	P	P	P	P	P	P	N	N	N	N	N	N	N	N	N	N	N	N	N	N	N	N	N	N	N	N	N	N	N	N	N	N
AK247	P	P	P	P	P	P	P	P	P	P	P	P	P	P	P	N	N	N	N	N	N	N	N	N	N	N	N	N	N	N	N	N	N	N	N	N	P	P	P	P	P	P	P	P	P	P	P	P	P	P
AMC35	P	P	P	P	P	P	P	P	P	P	P	P	P	P	P	N	N	N	N	N	N	N	N	N	N	N	N	N	N	N	N	N	N	N	N	N	N	N	N	N	N	N	N	N	N	N	N	N	N	N
CCM 4359	P	P	P	P	P	P	P	P	P	P	P	P	P	P	P	N	N	N	N	N	N	N	N	N	N	N	N	N	N	N	N	N	N	N	N	N	N	N	N	N	N	N	N	N	N	N	N	N	N	N
TTU2014-108AME	P	P	P	P	P	P	P	P	P	P	P	P	P	P	P	P	P	P	P	P	P	P	P	P	N	N	N	N	N	N	N	N	N	N	N	N	P	P	P	P	P	P	P	P	P	P	P	P	P	P
TTU2014-108ASC	P	P	P	P	P	P	P	P	P	P	P	P	P	P	P	P	P	P	P	P	P	P	P	P	N	N	N	N	N	N	N	N	N	N	N	N	P	P	P	P	P	P	P	P	P	P	P	P	P	P
TTU2014-113AME	P	P	P	P	P	P	P	P	P	P	P	P	P	P	P	P	P	P	P	P	P	P	P	P	P	P	P	N	P	P	P	P	P	P	P	P	P	P	P	P	P	P	P	P	P	P	P	P	P	P
TTU2014-115AME	P	P	P	P	P	P	P	P	P	P	P	P	P	P	P	P	P	P	P	P	P	P	P	P	N	N	N	N	N	N	N	N	N	N	N	N	P	P	P	P	P	P	P	P	P	P	P	P	P	P
TTU2014-115ASC	P	P	P	P	P	P	P	P	P	P	P	P	P	P	P	P	P	P	P	P	P	P	P	P	N	N	N	N	N	N	N	N	N	N	N	N	P	P	P	P	P	P	P	P	P	P	P	P	P	P
TTU2014-125ASC	P	P	P	P	P	P	P	P	P	P	P	P	P	P	P	P	P	P	P	P	P	P	P	P	P	P	P	N	P	P	P	P	P	P	P	P	P	P	P	P	P	P	P	P	P	P	P	P	P	P
TTU2014-130AME	P	P	P	P	P	P	P	P	P	P	P	P	P	P	P	P	P	P	P	P	P	P	P	P	P	P	P	N	P	P	P	P	P	P	P	P	P	P	P	P	P	P	P	P	P	P	P	P	P	P
TTU2014-130ASC	P	P	P	P	P	P	P	P	P	P	P	P	P	P	P	P	P	P	P	P	P	P	P	P	P	P	P	N	P	P	P	P	P	P	P	P	P	P	P	P	P	P	P	P	P	P	P	P	P	P
TTU2014-131ASC	P	P	P	P	P	P	P	P	P	P	P	P	P	P	P	P	P	P	P	P	P	P	P	P	P	P	P	N	P	P	P	P	P	P	P	P	P	P	P	P	P	P	P	P	P	P	P	P	P	P
TTU2014-134AME	P	P	P	P	P	P	P	P	P	P	P	P	P	P	P	P	P	P	P	P	P	P	P	P	P	P	P	N	P	P	P	P	P	P	P	P	P	P	P	P	P	P	P	P	P	P	P	P	P	P
TTU2014-134ASC	P	P	P	P	P	P	P	P	P	P	P	P	P	P	P	P	P	P	P	P	P	P	P	P	P	P	P	N	P	P	P	P	P	P	P	P	P	P	P	P	P	P	P	P	P	P	P	P	P	P
TTU2014-140ASC	P	P	P	P	P	P	P	P	P	P	P	P	P	P	P	P	P	P	P	P	P	P	P	P	P	P	P	N	P	P	P	P	P	P	P	P	P	P	P	P	P	P	P	P	P	P	P	P	P	P

TTU2014-141AME	P	P	P	P	P	P	P	P	P	P	P	P	P	P	P	P	P	P	P	P	P	P	P	P	P	P	P	N	P	P	P	P	P	P	P	P	P	P	P	P	P	P	P	P	P	P	P	P	P	P
TTU2014-141ASC	P	P	P	P	P	P	P	P	P	P	P	P	P	P	P	P	P	P	P	P	P	P	P	P	P	P	P	N	P	P	P	P	P	P	P	P	P	P	P	P	P	P	P	P	P	P	P	P	P	P
TTU2014-142ASC	P	P	P	P	P	P	P	P	P	P	P	P	P	P	P	P	P	P	P	P	P	P	P	P	P	P	P	N	P	P	P	P	P	P	P	P	P	P	P	P	P	P	P	P	P	P	P	P	P	P
TTU2014-143AME	P	P	P	P	P	P	P	P	P	P	P	P	P	P	P	P	P	P	P	P	P	P	P	P	P	P	P	N	P	P	P	P	P	P	P	P	P	P	P	P	P	P	P	P	P	P	P	P	P	P
TTU2014-143ASC	P	P	P	P	P	P	P	P	P	P	P	P	P	P	P	P	P	P	P	P	P	P	P	P	P	P	P	N	P	P	P	P	P	P	P	P	P	P	P	P	P	P	P	P	P	P	P	P	P	P
A31	P	P	P	P	P	P	P	N	P	P	P	P	P	P	P	P	P	P	P	P	P	P	P	P	N	N	N	N	N	N	N	N	N	N	N	N	N	N	N	N	N	N	N	N	N	N	N	N	N	N
A5	P	P	P	P	P	P	P	P	P	P	P	P	P	P	P	P	P	P	P	P	P	P	P	P	P	P	P	N	N	N	N	N	N	N	N	N	N	N	N	N	N	N	N	N	N	N	N	N	N	N
A86	P	P	P	P	P	P	P	P	P	P	P	P	P	P	P	P	P	P	P	P	P	P	P	P	N	N	N	N	N	N	N	N	N	N	N	N	N	N	N	N	N	N	N	N	N	N	N	N	N	N
A34	P	P	P	P	P	P	P	P	P	P	P	P	P	P	P	P	P	P	P	P	P	P	P	P	N	N	N	N	N	N	N	N	N	N	N	N	N	N	N	N	N	N	N	N	N	N	N	N	N	N
A136	P	P	P	P	P	P	P	P	P	P	P	P	P	P	P	P	P	P	P	P	P	P	P	P	N	N	N	N	N	N	N	N	N	N	N	N	N	N	N	N	N	N	N	N	N	N	N	N	N	N
Ae52	P	P	P	P	P	P	P	P	P	P	P	P	P	P	P	N	N	N	N	N	N	N	N	N	N	N	N	N	N	N	N	N	N	N	N	N	N	N	N	N	N	N	N	N	N	N	N	N	N	N
CL8155	P	P	P	P	P	P	P	P	P	P	P	P	P	P	P	P	P	P	P	P	P	P	P	P	N	N	N	N	N	N	N	N	N	N	N	N	P	P	P	P	N	P	P	P	P	P	P	P	P	P
JC529	P	P	P	P	P	P	P	P	P	P	P	P	P	P	P	P	P	P	P	P	P	P	P	P	N	N	N	N	N	N	N	N	N	N	N	N	P	P	P	P	P	P	P	P	P	P	P	P	P	P
MS 17-88	P	P	P	P	P	P	P	P	P	P	P	P	P	P	P	N	N	N	N	N	N	N	N	N	N	N	N	N	N	N	N	N	N	N	N	N	P	P	P	P	P	P	P	P	P	P	P	P	P	P
MS-18-37	P	P	P	P	P	P	P	P	P	P	P	P	P	P	P	N	N	N	N	N	N	N	N	N	N	N	N	N	N	N	N	N	N	N	N	N	N	N	N	N	N	N	N	N	N	N	N	N	N	N
ML09-123	P	P	P	P	P	P	P	P	P	P	P	P	P	P	P	P	P	P	P	P	P	P	P	P	N	N	N	N	N	N	N	N	N	N	N	N	P	P	P	P	P	P	P	P	P	P	P	P	P	P
VCK_1	P	P	P	P	P	P	P	P	P	P	P	P	P	P	P	P	P	P	P	P	P	P	P	P	N	N	N	N	N	N	N	N	N	N	N	N	P	P	P	P	P	P	P	P	P	P	P	P	P	P
PDB	P	P	P	P	P	P	P	P	P	P	P	P	P	P	P	P	P	P	P	P	P	P	P	P	N	N	N	N	N	N	N	N	N	N	N	N	N	N	N	N	N	N	N	N	N	N	N	N	N	N
AG_5.28.6	P	P	P	P	P	P	P	P	P	P	P	P	P	P	P	P	P	P	P	P	P	P	P	P	N	N	N	N	N	N	N	N	N	N	N	N	P	P	P	P	N	P	P	P	P	P	P	P	P	P
NS	P	P	P	P	P	P	P	P	P	P	P	P	P	P	P	P	P	P	P	P	P	P	P	P	N	N	N	N	N	N	N	N	N	N	N	N	N	N	N	N	N	N	N	N	N	N	N	N	N	N
NS2	P	P	P	P	P	P	P	P	P	P	P	P	P	P	P	P	P	P	P	P	P	P	P	P	N	N	N	N	N	N	N	N	N	N	N	N	N	N	N	N	N	N	N	N	N	N	N	N	N	N
NS_6.15.2	P	P	P	P	P	P	P	P	P	P	P	P	P	P	P	P	P	P	P	P	P	P	P	P	N	N	N	N	N	N	N	N	N	N	N	N	N	N	N	N	N	N	N	N	N	N	N	N	N	N
NS22	P	P	P	P	P	P	P	P	P	P	P	P	P	P	P	P	P	P	P	P	P	P	P	P	N	N	N	N	N	N	N	N	N	N	N	N	N	N	N	N	N	N	N	N	N	N	N	N	N	N
NS13	P	P	P	P	P	P	P	P	P	P	P	P	P	P	P	P	P	P	P	P	P	P	P	P	N	N	N	N	N	N	N	N	N	N	N	N	N	N	N	N	N	N	N	N	N	N	N	N	N	N
BIOO50A	P	P	P	P	P	P	P	P	P	P	P	P	P	P	P	P	P	P	P	P	P	P	P	P	N	N	N	N	N	N	N	N	N	N	N	N	P	P	P	P	N	P	P	P	P	P	P	P	P	P
17ISAe	P	P	P	P	P	P	P	P	P	P	P	P	P	P	P	P	P	P	P	P	P	P	P	P	N	N	N	N	N	N	N	N	N	N	N	N	N	N	N	N	N	N	N	N	N	N	N	N	N	N
A8-AHP	P	P	P	P	P	P	P	P	P	P	P	P	P	P	P	P	P	P	P	P	P	P	P	P	N	N	N	N	N	N	N	N	N	N	N	N	N	N	N	N	N	N	N	N	N	N	N	N	N	N
UBA1835	P	P	P	P	P	P	P	P	P	P	P	P	P	N	P	P	P	P	P	P	P	P	P	P	N	N	N	N	N	N	N	N	N	N	N	N	P	N	P	P	P	P	P	P	P	P	P	P	P	P
ZfB1	P	P	P	P	P	P	P	P	P	P	P	P	P	P	P	P	P	P	P	P	P	P	P	P	N	N	N	N	N	N	N	N	N	N	N	N	N	N	N	N	N	N	N	N	N	N	N	N	N	N
PhIn2	P	P	P	P	P	P	P	N	P	P	P	P	N	P	P	P	P	P	P	P	P	P	P	N	N	N	N	N	N	N	N	N	N	N	N	N	N	N	N	N	N	N	N	N	N	N	N	N	N	N
CB51	P	P	P	P	P	P	P	P	P	P	P	P	P	P	P	P	P	P	P	P	P	P	P	N	N	N	N	N	N	N	N	N	N	N	N	N	N	N	N	N	N	N	N	N	N	N	N	N	N	N
XH.VA.1	P	P	P	P	P	P	P	P	P	P	P	P	P	P	P	P	P	P	P	P	P	P	P	P	N	N	N	N	N	N	N	N	N	N	N	N	N	N	N	N	N	N	N	N	N	N	N	N	N	N
XH.VA.2	P	P	P	P	P	P	P	P	P	P	P	P	P	P	P	P	P	P	P	P	P	P	P	P	P	P	P	N	N	N	N	N	N	N	N	N	N	N	N	N	N	N	N	N	N	N	N	N	N	N
X11	P	P	P	P	P	P	P	P	P	P	P	P	P	P	P	N	N	N	N	N	N	N	N	N	N	N	N	N	N	N	N	N	N	N	N	N	N	N	N	N	N	N	N	N	N	N	N	N	N	N
X12	P	P	P	P	P	P	P	P	P	P	P	P	P	P	P	P	P	P	P	P	P	P	P	P	N	N	N	N	N	N	N	N	N	N	N	N	P	P	P	P	P	P	P	P	P	P	P	P	P	P
Aer_WatCTCBM21	P	P	P	P	P	P	P	P	P	P	P	P	P	P	P	P	P	P	P	P	P	P	P	P	N	N	N	N	N	N	N	N	N	N	N	N	N	N	N	N	N	N	N	N	N	N	N	N	N	N
CNRT12	P	P	P	P	P	P	P	P	P	P	P	P	P	P	P	P	P	P	P	P	P	P	P	P	N	N	N	N	N	N	N	N	N	N	N	N	N	N	N	N	N	N	N	N	N	N	N	N	N	N
NK01	P	P	P	P	P	P	P	P	P	P	P	P	P	P	P	P	P	P	P	P	P	P	P	P	N	N	N	N	N	N	N	N	N	N	N	N	N	N	N	N	N	N	N	N	N	N	N	N	N	N
NK02	P	P	P	P	P	P	P	P	P	P	P	P	P	P	P	N	P	P	P	P	P	P	P	P	P	P	P	N	N	N	N	N	N	N	N	N	N	N	N	N	N	N	N	N	N	N	N	N	N	N
NK07	P	P	P	P	P	P	P	P	P	P	P	P	P	P	P	P	P	P	P	P	P	P	P	P	N	N	N	N	N	N	N	N	N	N	N	N	N	N	N	N	N	N	N	N	N	N	N	N	N	N
UDRT09	P	P	P	P	P	P	P	P	P	P	P	P	P	P	P	P	P	P	P	P	P	P	P	P	N	N	N	N	N	N	N	N	N	N	N	N	N	N	N	N	N	N	N	N	N	N	N	N	N	N
BAQ071013-135	P	P	P	P	P	P	P	P	P	P	P	P	P	P	P	P	P	P	P	P	P	P	P	P	N	N	N	N	N	N	N	N	N	N	N	N	N	N	N	N	N	N	N	N	N	N	N	N	N	N

B44	P	P	P	P	P	P	P	P	P	P	P	P	P	P	P	P	P	P	P	P	P	P	P	P	P	P	N	P	P	P	N	P	P	P	P	P	P	P	P	P	P	P	P	P	P	P	P	P	P	P
B48	P	P	P	P	P	P	P	P	P	P	P	P	P	P	P	P	P	P	P	P	P	P	P	P	N	N	N	N	N	N	N	N	N	N	N	N	P	P	P	P	P	P	P	P	P	P	P	P	P	P
WB12	P	P	P	P	P	P	P	P	P	P	P	P	P	P	P	N	N	N	N	N	N	N	N	N	N	N	N	N	N	N	N	N	N	N	N	N	N	N	N	N	N	N	N	N	N	N	N	N	N	N
AVNIH1 (GCA_009834065)	P	P	P	P	P	P	P	P	P	P	P	P	P	P	P	N	P	P	P	P	P	P	P	P	N	N	N	N	N	N	N	N	N	N	N	N	N	N	N	N	N	N	N	N	N	N	N	N	N	N
TH0426	P	P	P	P	P	P	P	P	P	P	P	P	P	P	P	P	P	P	P	P	P	P	P	P	N	N	N	N	N	N	N	N	N	N	N	N	P	P	P	P	P	P	P	P	P	P	P	P	P	P
XU1	P	P	P	P	P	P	P	P	P	P	P	P	P	P	P	N	N	N	N	N	N	N	N	N	P	P	P	N	N	N	N	N	N	N	N	N	N	N	N	N	N	N	N	N	N	N	N	N	N	N
XhG1.2	P	P	P	P	P	P	P	P	P	P	P	P	P	P	P	N	N	N	N	N	N	N	N	N	N	N	N	N	N	N	N	N	N	N	N	N	N	N	N	N	N	N	N	N	N	N	N	N	N	N
HX3	P	P	P	P	P	P	P	P	P	P	P	P	P	P	P	N	N	N	N	N	N	N	N	N	P	P	P	N	N	N	N	N	N	N	N	N	N	N	N	N	N	N	N	N	N	N	N	N	N	N
CQ-AV1	P	P	P	P	P	P	P	P	P	P	P	P	P	P	P	P	P	P	P	P	P	P	P	P	P	P	P	N	N	N	N	N	N	N	N	N	N	N	N	N	N	N	N	N	N	N	N	N	N	N
161	P	P	P	P	P	P	P	P	P	P	P	P	P	P	P	P	P	P	P	P	P	P	P	P	N	N	N	N	N	N	N	N	N	N	N	N	P	P	P	P	P	P	P	P	P	P	P	P	P	P
LMG 13067	P	P	P	P	P	P	P	P	P	P	P	P	P	P	P	N	N	N	N	N	N	N	N	N	N	N	N	N	N	N	N	N	N	N	N	N	N	N	N	N	N	N	N	N	N	N	N	N	N	N
S00030	P	P	P	P	P	P	P	P	P	P	P	P	P	P	P	P	P	P	P	P	P	P	P	P	N	N	N	N	N	N	N	N	N	N	N	N	N	N	N	N	N	N	N	N	N	N	N	N	N	N
Hm21	P	P	P	P	P	P	P	P	P	P	P	P	P	P	P	P	P	P	P	P	P	P	P	P	N	N	N	N	N	N	N	N	N	N	N	N	P	P	P	P	P	P	P	P	P	P	P	P	P	P
CMF	P	P	P	P	P	P	P	P	P	P	P	P	P	P	P	N	P	P	P	P	P	P	P	P	N	N	N	N	N	N	N	N	N	N	N	N	N	N	N	N	N	N	N	N	N	N	N	N	N	N
CIP 107763	P	P	P	P	P	P	P	P	P	P	P	P	P	P	P	P	P	P	P	P	P	P	P	P	P	P	P	P	P	N	P	P	N	P	P	P	N	N	N	N	N	N	N	N	N	N	N	N	N	N
AK241	P	P	P	P	P	P	P	P	P	P	P	P	P	P	P	P	P	P	P	P	P	P	P	P	N	N	N	N	N	N	N	N	N	N	N	N	P	P	P	P	P	P	P	P	P	P	P	P	P	P
B565	P	P	P	P	P	P	P	P	P	P	P	P	P	P	P	N	N	N	N	N	N	N	N	N	N	N	N	N	N	N	N	N	N	N	N	N	N	N	N	N	N	N	N	N	N	N	N	N	N	N
22	P	P	P	P	P	P	P	P	P	P	P	P	P	P	P	N	N	N	N	N	N	N	N	N	P	P	P	N	N	N	N	N	N	N	N	N	N	N	N	N	N	N	N	N	N	N	N	N	N	N
28	P	P	P	P	P	P	P	P	P	P	P	P	P	P	P	N	N	N	N	N	N	N	N	N	P	P	P	N	N	N	N	N	N	N	N	N	N	N	N	N	N	N	N	N	N	N	N	N	N	N
CECT 7059	P	P	P	P	P	P	P	P	P	P	P	P	P	P	P	P	P	P	P	P	P	P	P	P	N	N	N	N	N	N	N	N	N	N	N	N	P	P	P	P	P	P	P	P	P	P	P	P	P	P
RU31B	P	P	P	P	P	P	P	P	P	P	P	P	P	P	P	P	P	P	P	P	P	P	P	P	N	N	N	N	N	N	N	N	N	N	N	N	P	P	P	P	P	P	P	P	P	P	P	P	P	P
CECT 4902	P	P	P	P	P	P	P	P	P	P	P	P	P	P	P	P	P	P	P	P	P	P	P	P	N	N	N	N	N	N	N	N	N	N	N	N	N	N	N	N	N	N	N	N	N	N	N	N	N	N
AK236	P	P	P	P	P	P	P	P	P	P	P	P	P	P	P	N	N	N	N	N	N	N	N	N	N	N	N	N	N	N	N	N	N	N	N	N	N	N	N	N	N	N	N	N	N	N	N	N	N	N
Colony604	P	P	P	P	P	P	P	N	P	P	N	P	P	P	P	N	N	N	N	N	N	N	N	N	N	N	N	N	N	N	N	N	N	N	N	N	N	N	N	N	N	N	N	N	N	N	N	N	N	N
Colony111	P	P	P	P	P	P	P	N	P	P	N	P	P	P	P	N	N	N	N	N	N	N	N	N	N	N	N	N	N	N	N	N	N	N	N	N	N	N	N	N	N	N	N	N	N	N	N	N	N	N
Colony512	P	P	P	P	P	P	P	P	P	P	P	P	P	P	P	N	N	N	N	N	N	N	N	N	N	N	N	N	N	N	N	N	N	N	N	N	N	N	N	N	N	N	N	N	N	N	N	N	N	N
Colony125	P	P	P	P	P	P	P	N	P	P	N	P	P	P	P	N	N	N	N	N	N	N	N	N	N	N	N	N	N	N	N	N	N	N	N	N	N	N	N	N	N	N	N	N	N	N	N	N	N	N
pamvotica	P	P	P	P	P	P	P	P	P	P	P	P	P	P	P	P	P	P	P	P	P	P	P	P	P	P	P	P	P	N	N	P	N	P	P	P	P	P	P	P	P	P	P	P	P	P	P	P	P	P
A134	P	P	P	P	P	P	P	P	P	P	P	P	P	P	P	N	N	N	N	N	N	N	N	N	N	N	N	N	N	N	N	N	N	N	N	N	N	N	N	N	N	N	N	N	N	N	N	N	N	N
S50-1	P	P	P	P	P	P	P	P	P	P	P	P	P	P	P	P	P	P	P	P	P	P	P	P	N	N	N	N	N	N	N	N	N	N	N	N	N	N	N	N	N	N	N	N	N	N	N	N	N	N
CTe-01	P	P	P	P	P	P	P	P	P	P	P	P	P	P	P	P	P	P	P	P	P	P	P	P	N	N	N	N	N	N	N	N	N	N	N	N	N	N	N	N	N	N	N	N	N	N	N	N	N	N
ARB3	P	P	P	P	P	P	P	P	P	P	P	P	P	P	P	P	P	P	P	P	P	P	P	P	N	N	N	N	N	N	N	N	N	N	N	N	P	P	P	P	P	P	P	P	P	P	P	P	P	P
Z2-7	P	P	P	P	P	P	P	P	P	P	P	P	P	P	P	N	N	N	N	N	N	N	N	N	N	N	N	N	N	N	N	N	N	N	N	N	N	N	N	N	N	N	N	N	N	N	N	N	N	N
KLG7	P	P	P	P	P	P	P	P	P	P	P	P	P	P	P	P	P	P	P	P	P	P	P	P	N	N	N	N	N	N	N	N	N	N	N	N	P	P	P	P	P	P	P	P	P	P	P	P	P	P
KLG5	P	P	P	P	P	P	P	P	P	P	P	P	P	P	P	P	P	P	P	P	P	P	P	P	N	N	N	N	N	N	N	N	N	N	N	N	P	P	P	P	P	P	P	P	P	P	P	P	P	P
KLG8	P	P	P	P	P	P	P	P	P	P	P	P	P	P	P	N	P	P	P	P	P	P	P	P	N	N	N	N	N	N	N	N	N	N	N	N	P	P	P	P	P	P	P	P	P	P	P	P	P	P
KLG9	P	P	P	P	P	P	P	P	P	P	P	P	P	P	P	P	P	P	P	P	P	P	P	P	N	N	N	N	N	N	N	N	N	N	N	N	P	P	P	P	P	P	P	P	P	P	P	P	P	P
CECT 4486	P	P	P	P	P	P	P	P	P	P	P	P	P	P	P	P	P	P	P	P	P	P	P	P	N	N	N	N	N	N	N	N	N	N	N	N	P	P	P	P	P	P	P	P	P	N	P	P	P	P
CCM 7244	P	P	P	P	P	P	P	P	P	P	P	P	P	P	P	P	P	P	P	P	P	P	P	P	N	N	N	N	N	N	N	N	N	N	N	N	P	P	P	P	P	P	P	P	P	P	P	P	P	P
A29	P	P	P	P	P	P	P	P	P	P	P	P	P	P	P	P	P	P	P	P	P	P	P	P	N	N	N	N	N	N	N	N	N	N	N	N	N	N	N	N	N	N	N	N	N	N	N	N	N	N
AK227	P	P	P	P	P	P	P	P	P	P	P	P	P	P	P	N	N	N	N	N	N	N	N	N	N	N	N	N	N	N	N	N	N	N	N	N	N	N	N	N	N	N	N	N	N	N	N	N	N	N
WP2-S18-CRE-03	P	P	P	P	P	P	P	P	P	P	P	P	P	P	P	N	N	N	N	N	N	N	N	N	N	N	N	N	N	N	N	N	N	N	N	N	N	N	N	N	N	N	N	N	N	N	N	N	N	N

WP3-W19-ESBL-03	P	P	P	P	P	P	P	P	P	P	P	P	P	P	P	N	P	P	P	P	P	P	P	P	N	N	N	N	N	N	N	N	N	N	N	N	N	N	N	N	N	N	N	N	N	N	N	N	N	N
WP8-S18-ESBL-11	P	P	P	P	P	P	P	P	P	P	P	P	P	P	P	N	N	N	N	N	N	N	N	N	N	N	N	N	N	N	N	N	N	N	N	N	N	N	N	N	N	N	N	N	N	N	N	N	N	N
WP8-W19-CRE-03	P	P	P	P	P	P	P	P	P	P	P	P	P	P	P	N	N	N	N	N	N	N	N	N	N	N	N	N	N	N	N	N	N	N	N	N	N	N	N	N	N	N	N	N	N	N	N	N	N	N
WP9-W18-ESBL-04	P	P	P	P	P	P	P	P	P	P	P	P	P	P	P	N	N	N	N	N	N	N	N	N	P	P	P	N	N	N	N	N	N	N	N	N	N	N	N	N	N	N	N	N	N	N	N	N	N	N
D	P	P	P	P	P	P	P	P	P	P	P	P	P	P	P	P	P	P	P	P	P	P	P	P	N	N	N	N	N	N	N	N	N	N	N	N	P	P	P	P	N	P	P	P	P	P	P	P	P	P

Additional file 1 The prevalence of bacterial secretion systems in 168 *Aeromonas veronii* strains

There are two different strains of which both named AVNIH1, the corresponding accession numbers are indicated in brackets.
