# Supplementary material for: Analysis of global Aeromonas veronii genomes provides novel information on source of infection and virulence in human gastrointestinal diseases
Source: BMC Genomics. 2022 Feb 28;23:166. doi: 10.1186/s12864-022-08402-1 (PMC8883699; doi:10.1186/s12864-022-08402-1)
Supplement: Supplementary file 2 — Additional file 2. [file 12864_2022_8402_MOESM2_ESM.pdf]

```
A20                    -MNRIITANLAFLASSLMLAQVQAAEPVYPDQVKWAGLGTGVCASGYRPLTRDEAMSIKG   59
A20-10                 MMNRIITANLAFLASSLMLAQVQAAEPVYPDQVKWAGLGTGVCASGYRPLTRDEAMSIKG   60
A20-12                 MMNRIITANLAFLASSLMLAQVQAAEPVYPDQVKWAGLGTGLCASGYRPLTRDEAMSIKG   60
A20-14                 MMNRIITANLAFLASSLMLAQVQAAEPVYPDQVKWAGLGTGVCASGYRPLTRDEAMSIKG   60
A20-17                 MMNRIITANLAFLASSLMLAQVQAAEPVYPDQVKWAGLGTGVCASGYRPLTRDEAMSIKG   60
A20-5                  MMNRIITANLAFLASSLMLAQVQAAEPVYPDQVKWAGLGTGVCASGYRPLTRDEAMSIKG   60
A20-8                  MMNRIITANLAFLASSLMLAQVQAAEPVYPDQVKWAGLGTGVCASGYRPLTRDEAMSIKG   60
A21                    MMNRIITANLAFLASSLMLAQVQAAEPVYPDQVKWAGLGTGVCASGYRPLTRDEAMSIKG   60
A21-10                 MMNRIITANLAFLASSLMLAQVQAAEPVYPDQVKWAGLGTGVCASGYRPLTRDEAMSIKG   60
A21-11                 MMNRIITANLAFLASSLMLAQVQAAEPVYPDQVKWAGLGTGLCASGYRPLTRDEAMSIKG   60
A21-13                 MMNRIITANLAFLASSLMLAQVQAAEPVYPDQVKWAGLGTGVCASGYRPLTRDEAMSIKG   60
A21-14                 -MNKVISVNIILLSTIFMLMKVQAAEPIYPAQVKWYGLGQGVCANGYRPLTRDEAMSIKN   59
A21-15                 MMNRIIIANLAFLASSLMLAQVQAAEPVYPDQVKWAGLGTGVCASGYRPLTRDEAMSIKG   60
A21-16                 MMNRIITANLAFLASSLMLAQVQAAEPVYPDQVKWAGLGSGVCASGYRPLTRDEAMSIKG   60
A21-19                 MMNKLITANLALFASSLMLIQAQAAEPVYPDQVKWYGLGEGVCASGYRPLTRDEAMSIKG   60
A21-4                  MMNRIITANLAFLASSLMLAQVQAAEPVYPDQVKWAGLGTGVCSSGYRPLTRDEAMSIKG   60
A21-5                  MMNRIITANLAFLASSLMLAQVQAAEPVYPDQVKLAGLGTGVCASGYRPLTRDEAMSIKG   60
A21-6                  MMNRIITANLAFLASSLMLAQVQAAEPVYPDQVKWAGLGTDVCASGYRPLTRDEAMSIKD   60
A21-8                  MMNRIITASLAFLASSLMLAQVQAAEPVYPDQVKWAGLGTGVCASGYRPLTRDEAMSIKG   60
A26                    MMNRIITANLAFLASSLMLAQVQAAEPVYPDQVKWAGLGTGVCASGYRPLTRDEAMSIKG   60
A27                    MMNRIITANLAFWASSLMLAQVQAAEPVYPDQVKWAGLGTGVCASGYRPLTRDEAMSIKG   60
A29V                   MMNRIITANLAFLASSLMLAQVQAAEPVYPDQVKWAGLGTGVCASGYRPLTRDEAMSIKG   60
A7                     MMNRIITANLAFLASSLMLAQVQAAEPVYPDQVKWAGLGTGVCASGYRPLTRDEAMSIKG   60
A8                     MMNRIITANLAFLASSLMLAQVQAAEPVYPDQVKWAGLGTGVCASGYRPLTRDEAMSIKS   60
A9                     MMNRIITANLAFLASSLMLAQVQAAEPVYPDQVKWAGLGTGVCSSGYRPLTRDEAMSIKG   60
BC88                   -MNRIITANLAFLASSLMLAQVQAAEPVYPDQVKWAGLGTGVCASGYRPLTRDEAMSIKG   59
FC951                  ------------------------------------------------------------   0
126-14                 MMNRIITANLAFLASSLMLAQVQAAEPVYPDQVKWASLGTGVCASGYRPLTRDEAMSIKG   60
312M                   MMNRIITANLAFLASSLMLAQVQAAEPVYPDQVKWAGLGTGVCASGYRPLTRDEAMSIKG   60
VBF557                 MMNRIITANLAFLASSLMLAQVQAAEPVYPDQVKWAGLGTGVCASGYRPLTRDEAMSIKG   60
ERR1305902-bin.15      MMNRIITANLAFLASSLMLAQVQAAEPVYPDQVKWASLGTGVCASGYRPLTRDEAMSIKG   60


A20                    NLVSRMGQWQITGLADRWVIMGPGYNGEIKQG--TAGETWCYPNSPVSGEIPTLSDWNIP   117
A20-10                 NLVSRMGQWQITGLADRWVIMGSGYNGEIKQG--TAGETWCYPNSPVSGEIPTLSDWNIP   118
A20-12                 NLVSRMGQWQITGLADRWVIMGPGYNGEIKQG--TAGETWCYPNSPVSGEIPTLSDWNIP   118
A20-14                 NLVSRMGQWQITGLADRWVIMGPGYNGEIKQG--TAGETWCYPNSPVSGEIPTLSDWNIP   118
A20-17                 NLVSRMGQWQITGLADRWVIMGPGYNGEIKQG--TAGETWCYPNSPVSGEIPTLSDWNIP   118
A20-5                  NLVSRMGQWQITGLADRWVIMGPGYNGEIKQG--TAGETWCYPNSPVSGEIPTLSDWNIP   118
A20-8                  NLVSRMGQWQITGLADRWVIMGPGYNGEIKQG--TAGETWCYPNSPVSGEIPTLSDWNIP   118
A21                    NLVSRMGQWQITGLADRWVIMGPGYNGEIKQG--TAGETWCYPNSPVSGEIPTLSDWNIP   118
A21-10                 NLVSRMGQWQITGLADRWVIMGPGYNGEIKQG--TAGETWCYPNSPVSGEIPTLSDWNIP   118
A21-11                 NLVSRMGQWQITGLADRWVIMGPGYNGEIKQG--TAGETWCYPNSPVSGEIPTLSDWNIP   118
A21-13                 NLVSRMGQWQIAGLADRWVIMGPGYNGEIKQG--TAGETWCYPNSPVSGEIPTLSDWNIP   118
A21-14                 SLVSRMGPWQITGLADGWVIMGPGYNGEIKKSLVSAGESWCYPNSPVSGEIPTLSDWNIP   119
A21-15                 NLVSRMGQWQITGLAERWVIMGPGYNGEIKQG--TAGETWCYPNSPVSGEIPTLSDWNIP   118
A21-16                 NLVSRMGQWQITGLADRWVIMGPGYNGEIKQG--TAGETWCYPNSPVSGEIPTLSDWNIP   118
A21-19                 NLVGRMGQWQITGLANRWVIMGSGYNGEIKQG--TAGETWCYPTSPISGEIPSLSAWNIP   118
A21-4                  NLVSRMGQWQITGLADRWVIMGSGYNGEIKQG--TAGETWCYPNSPVSGEIPTLSDWNIP   118
A21-5                  NLVSRMGQWQITGLADRWVIMGPGYNGEIKQG--TAGETWCYPNSPVSGEIPTLSDWNIP   118
A21-6                  NLVSRMGQWQITGLANRWVIMGPGYNGEIKQG--SAGETWCYPNSPVSGEIPTLSVWNIP   118
A21-8                  NLVSRMGQWQITGLADRWVIMGPGYNGEIKQG--TAGETWCYPNSPVSGEIPTLSDWNIP   118
A26                    NLVSRMGQWQITGLADRWVIMGPGYNGEIKQG--TAGETWCYPNSPVSGEIPTLSDWNIP   118
A27                    NLVSRMGQWQITGLADRWVIMGPGYNGEIKQG--TAGETWCYPNSPVSGEIPTLSDWNIP   118
A29V                   NLVSRMGQWQITGLADRWVIMGPGYNGEIKQG--TAGETWCYPNSPVSGEIPTLSDWNIP   118
A7                     NLVSRMGQWQITGLADRWVIMGPGYNGEIKQG--TAGETWCYPNSPVSGEIPTLSDWNIP   118
A8                     NLVSRMGQWQITGLADRWVIMGPGYNGEIKQG--TAGETWCYPNSPVSGEIPTLSDWNIP   118
A9                     NLVSRMGQWQITGLADRWVIMGSGYNGEIKQG--TAGETWCYPNSPVSGEIPTLSDWNIP   118
BC88                   NLVSRMGQWQITGLADRWVIMGSGYNGEIKQG--TAGETWCYPNSPVSGEIPTLSDWNIP   117
FC951                  ------------------------------------------------------------   0
126-14                 NLVSRMGQWQITGLADRWVIMGSGYNGEIKQG--TAGETWCYPNSPVSGEIPTLSDWNIP   118
312M                   NLVSRMGQWQITGLADRWVIMGPGYNGEIKQG--TAGETWCYPNSPVSGEIPTLSDWNIP   118
VBF557                 NLVSRMGQWQITGLADRWVIMGPGYNGEIKQG--TAGETWCYPNSPVSGEIPTLSDWNIP   118
ERR1305902-bin.15      NLVSRMGQWQITGLADRWVIMGPGYNGEIKQG--TAGETWCYPNSPVSGEIPTLSDWNIP   118
```

```
A20                    AGDEVDVQWRLVHDNDYFIKPVSYLAHYLGYAWVGGNHSPYVGEDMDVTRVGDGWLIKGN   177
A20-10                 AGDEVDVQWRLVHDNDYFIKPVSYLAHYLGYAWVGGNHSPYVGEDMDVTRVGDGWLIKGN   178
A20-12                 AGDEVDVQWRLVHDNDYFIKPVSYLAHYLGYAWVGGNHSPYVGEDMDVTRVGDGWLIKGN   178
A20-14                 AGDEVDVQWRLVHDNDYFIKPVSYLAHYLGYAWVGGNHSPYVGEDMDVTRVGDGWLIKGN   178
A20-17                 AGDEVDVQWRLVHDNHYFIKPVSYLAHYLGYAWVGGNHSPYVGEDMDVTRVGDTWLIKGN   178
A20-5                  AGDEVDVQWRLVHDNNYFIKPVSYLAHYLGYAWVGGNHSPYVGEDMDVTRVGDGWLIKGN   178
A20-8                  AGDEVDVQWRLVHDNDYFIKPVSYLAHYLGYAWVGGNHSPYVGEDMDVTRVGDGWLIKGN   178
A21                    AGDEVDVQWRLVHDNDYFIKPVSYLAHYLGYAWVGGNHSPYVGEDMDVTRVGDGWLIKGN   178
A21-10                 AGDEVDVQWRLVHDNDYFIKPVSYLAHYLGYAWVGGNHSPYVGEDMDVTRVGDGWLIKGN   178
A21-11                 AGDEVDVQWRLVHDNDYFIKPVSYLAHYLGYAWVGGNHSPYVGEDMDVTRVGDGWLIKGN   178
A21-13                 AGDEVDVQWRLVHDNDYFIKPVSYLAHYLGYAWVGGNHSPYVGEDMDVTRVGDGWLIKGN   178
A21-14                 AGDEVDVQWRLVHDNDYFIKPVSYLAYFLGYARVGGNHSPYVGEDMDVTRLSDGWLIKGN   179
A21-15                 AGDEVDVQWRLVHDNDYFIKPVSYLAHYLGYAWVGGNHSPYVGEDMDVTRVGDGWLIKGN   178
A21-16                 AGDEVDVQWRLVHDNDYFIKPVSYLAHYLGYAWVGGNHSPYVGEDMDVTRVGDGWLIKGN   178
A21-19                 AGDEVDVQWRMVHDNDYFIKPVSYLAHYLGYAWVGGNHSPYVGEDMDVTRLSDGWLIKGN   178
A21-4                  AGDEVDVQWRLVHDNDYFIKPVSYLAHYLGYAWVGGNHSPYVGEDMDVTRVGDGWLIKGN   178
A21-5                  AGDEVDVQWRLVHDNDYFIKPVSYLAHYLGYAWVGGNHSPYVGEDMDVTRVGDGWLIKGN   178
A21-6                  AGDEVDVQWRLVHDNDYFIKPASYLAHYLGYAWVGGNHSPYVGEDMDVTRVGDTWLIKGN   178
A21-8                  AGDEVDVQWRLVHDNDYFIKPVSYLAHYLGYAWVGGNHSPYVGEDMDVTRVGDGWLIKGN   178
A26                    AGDEVDVQWRLVHDNDYFIKPVSYLAHYLGYAWVGGNHSPYVGEDMDVTRVGDGWLIRGN   178
A27                    AGDEVDVQWRLVHDNDYFIKPVSYLAHYLGYAWVGGNHSPYVGEDMDVTRVGDGWLIKGN   178
A29V                   AGDEVDVQWRLVHDNDYFIKPVSYLAHYLGYAWVGGNHSPYVGEDMDVTRVGDGWLIKGN   178
A7                     AGDEVDVQWRLVHDNDYFIKPVSYLAHYLGYAWVGGNHSPYVGEDMDVTRVGDGWLIKGN   178
A8                     AGDEVDVQWRLVHDNDYFIKPVSYLAHYLGYAWVGGNHSPYVGEDMDVTRVGDGWLIKGN   178
A9                     AGDEVDVQWRLVHDNDYFIKPVSYLAHYLGYAWVGGNHSPYVGEDMDVTRVGDGWLIKGN   178
BC88                   AGDEVDVQWRLVHDNDYFIKPVSYLAHYLGYAWVGGNHSPYVGEDMDVTRVGDGWLIKGN   177
FC951                  ------MQWRLVHDNDYFIKPVSYLAHYLGYAWVGGNHSPYVGEDMDVTRVGDGWLIKGN   54
126-14                 AGDEVDVQWRLVHDNDYFIKPVSYLAHYLGYAWVGGNHSPYVGEDMDVTRVGDGWLIKGN   178
312M                   AGDEVDVQWRLVHDNDYFIKPVSYLAHYLGYAWVGGNHSPYVGEDMDVTRVGDGWLIKGN   178
VBF557                 AGDEVDVQWRLVHDNDYFIKPVSYLAHYLGYAWVGGNHSPYVGEDMDVTRVGDGWLIKGN   178
ERR1305902-bin.15      AGDEVDVQWRLVHDNDYFIKPVSYLAHYLGYAWVGGNHSPYVGEDMDVTRVGDGWLIKGN   178
                             :***:****.*****.****::**** *****************:.* ***:**


A20                    NDGGCSGYRCGEKSSIKVSNFSYTLEPDSFSHGQVTESGKQLVKTITANATNYTDLPQQV   237
A20-10                 NDGGCSGYRCGEKSSIKVSNFSYTLEPDSFSHGQVTESGKQLVKTITANATNYTDLPQQV   238
A20-12                 NDGGCSGYRCGEKSSIKVSNFSYTLEPDSFSHGQVTESGKQLVKTITANATNYTDLPQQV   238
A20-14                 NDGGCSGYRCGEKSSIKVSNFSYTLEPDSFSHGQVTESGKQLVKTITANATNYTDLPQQV   238
A20-17                 NDGGCSGYRCGEKSSITVSNFSYTLEPDSFSHGQVTESGKQLVKTITANATNYTDLPQQV   238
A20-5                  NDGGCSGYRCGEKSSIKVSNFSYTLEPDSFSHGQVTESGKQLVKTITANATNYTDLPQQV   238
A20-8                  NDGGCSGYRCGEKSSIKVSNFSYTLEPDSFSHGQVTESGKQLVKTITANATNYTDLPQQV   238
A21                    NDGGCSGYRCGEKSSIKVSNFSYTLEPDSFSHGQVTESGKQLVKTITANATNYTDLPQQV   238
A21-10                 NDGGCSGYRCGEKSSIKVSNFSYTLEPDSFSHGQVTESGKQLVKTITANATNYTDLPQQV   238
A21-11                 NDGGCSGYRCGEKSSIKVSNFSYTLEPDSFSHGQVTESGKQLVKTITANATNYTDLPQQV   238
A21-13                 NDGGCSGYRCGEKSSIKVSNFSYTLEPDSFSHGQVTESGKQLVKTITANATNYTDLPQQV   238
A21-14                 NDGGCSGYRCGEKSSIKVSNFSYTLDPDSFSHGQVTESGKQLVKNITANAINNTDQPQQV   239
A21-15                 NDGGCSGYRCGEKSSIKVSNFSYTLEPDSFSHGQVTESGKQLVKTITANATNYTDLPQQV   238
A21-16                 NDGGCSGYRCGEKSSIKVSNFSYTLEPDSFSHGQVTESGKQLVKTITANATNYTDLPQQV   238
A21-19                 NDGGCSGYRCGEKSSIKVSNFSYTLEPDSFTHGQVTESGKQLVKMITANATNYTDLPQQV   238
A21-4                  NDGGCSGYRCGEKSSIKVSNFSYTLEPDSFSHGQVTESGKQLVKTITANATNYTDLPQQV   238
A21-5                  NDGGCSGYRCGEKSSIKVSNFSYTLEPDSFSHGQVTESGKQLVKTITANATNYTDLPQQV   238
A21-6                  NDGGCSGYRCGEKSSITVSNFSYTLEPDSFSHGQVTESGKQLVKTITANATNYTDLPQQV   238
A21-8                  NDGGCSGYRCGEKSSIKVSNFSYTLEPDSFSHGQVTESGKQLVKTITANATNYTDLPQQV   238
A26                    NDGGCSGYRCGEKSSIKVSNFSYTLEPDSFSHGQVTESGKQLVKTITANATNYTDLPQQV   238
A27                    NDGGCSGYRCGEKSSIKVSNFSYTLEPDSFSHGQVTESGKQLVKTITANATNYTDLPQQV   238
A29V                   NDGGCSGYRCGEKSSIKVSNFSYTLEPDSFSHGQVTESGKQLVKTITANATNYTDLPQQV   238
A7                     NDGGCSGYRCGEKSSIKVSNFSYTLEPDSFSHGQVTESGKQLVKTITANATNYTDLPQQV   238
A8                     NDGGCSGYRCGEKSSIKVSNFSYTLEPDSFSHGQVTESGKQLVKTITANATNYTDLPQQV   238
A9                     NDGGCSGYRCGEKSSIKVSNFSYTLEPDSFSHGQVTESGKQLVKTITANATNYTDLPQQV   238
BC88                   NDGGCSGYRCGEKSSIKVSNFSYTLEPDSFSHGQVTESGKQLVKTITANATNYTDLPQQV   237
FC951                  NDGGCSGYRCGEKSSIKVSNFSYTLEPDSFSHGQVTESGKQLVKTITANATNYTDLPQQV   114
126-14                 NDGGCSGYRCGEKSSIKVSNFSYTLEPDSFSHGQVTESGKQLVKTITANATNYTDLPQQV   238
312M                   NDGGCSGYRCGEKSSIKVSNFSYTLEPDSFSHGQVTESGKQLVKTITANATNYTDLPQQV   238
VBF557                 NDGGCSGYRCGEKSSIKVSNFSYTLEPDSFSHGQVTESGKQLVKTITANATNYTDLPQQV   238
ERR1305902-bin.15      NDGGCSGYRCGEKSSIKVSNFSYTLEPDSFSHGQVTESGKQLVKTITANATNYTDLPQQV   238
                       ****************.********:****:************* ***** * ** ****
```

```
A20                   VVTLKYDKATNWSKTDTYSLSEKVTTKNKFQWPLVGETELAIEIAASQSWASQHGGSTTE   297
A20-10                VVTLKYDKATNWSKTDTYSLSEKVTTKNKFQWPLVGETELAIEIAASQSWASQKGGSTTE   298
A20-12                VVTLKYDKATNWSKTDTYSLSEKVTTKNKFQWPLVGETELAIEIAASQSWASQKGGSTTE   298
A20-14                VVTLKYDKATNWSKTDTYSLSEKVTTKNKFQWPLVGETELAIEIAASQSWASQKGGSTTE   298
A20-17                VVTLKYDKATNWSKTDTYSLSEKVTTKNKFQWPLVGETELAIEIAASQSWASQHGGSTTE   298
A20-5                 VVTLKYDKATNWSKTDTYSLSEKVTTKNKFQWPLVGETELAIEIAASQSWASQKGGSTTE   298
A20-8                 VVTLKYDKATNWSKTDTYSLSEKVTTKNKFQWPLVGETELAIEIAASQSWASQKGGSTTE   298
A21                   VVTLKYDKATNWSKTDTYSLSEKVTTKNKFQWPLVGETELAIEIAASQSWASQKGGSTTE   298
A21-10                VVTLKYDKATNWSKTDTYSLSEKVTTKNKFQWPLVGETELAIEIAASQSWASQKGGSTTE   298
A21-11                VVTLKYDKATNWSKTDTYSLSEKVTTKNKFQWPLVGETELAIEIAASQSWASQKGGSTTE   298
A21-13                VVTLKYDKATNWSKTDTYSLSEKVTTKNKFQWPLVGETELAIEIAASQSWASQKGGSTTE   298
A21-14                VVTLKYDKSTKWSKTDTYSLGELVEIKTSFQIPQVSKTEYSVEISSTQSWASQQGEETRE   299
A21-15                VVTLKYDKATNWSKTDTYSLSEKVTTKNKFQWPLVGETELAIEIAASQSWASQKGGSTTE   298
A21-16                VVTLKYDKATNWSKTDTYSLSEKVTTKNKFQWPLVGETELAIEIAASQSWASQKGGSTTE   298
A21-19                VVTLKYDKATNWSKTDTYSLSEKVGIKKTFQIPQVSSTEYSVEISSTQSWAEQKGGSTTE   298
A21-4                 VVTLKYDKATNWSKTDTYSLSEKVTTKNKFQWPLVGETELAIEIAASQSWASQKGGSTTE   298
A21-5                 VVTLKYDKATNWSKTDTYSLSEKVTTKNKFQWPLVGETELAIEIAASQSWASQKGGSTTE   298
A21-6                 VVTLKYDKATNWSKTDTYSLSEKVTTKNKFQWPLVGETELAIEIAASQSWASQHGGSTTE   298
A21-8                 VVTLKYDKATNWSKTDTYSLSEKVTTKNKFQWPLVGETELAIEIAASQSWASQKGGSTTE   298
A26                   VVTLKYDKATNWSKTDTYSLSEKVTTKNKFQWPLVGETELAIEIAASQSWASQHGGSTTE   298
A27                   VVTLKYDKATNWSKTDTYSLSEKVTTKNKFQWPLVGETELAIEIAASQSWASQKGGSTTE   298
A29V                  VVTLKYDKATNWSKTDTYSLSEKVTTKNKFQWPLVGETELAIEIAASQSWASQKGGSTTE   298
A7                    VVTLKYDKATNWSKTDTYSLSEKVTTKNKFQWPLVGETELAIEIAASQSWASQKGGSTTE   298
A8                    VVTLKYDKATNWSKTDTYSLSEKVTTKNKFQWPLVGETELAIEIAASQSWASQKGGSTTE   298
A9                    VVTLKYDKATNWSKTDTYSLSEKVTTKNKFQWPLVGETELAIEIAASQSWASQKGGSTTE   298
BC88                  VVTLKYDKATNWSKTDTYSLSEKVTTKNKFQWPLVGETELAIEIAASQSWASQKGGSTTE   297
FC951                 VVTLKYDKATNWSKTDTYSLSEKVTTKNKFQWPLVGETELAIEIAASQSWASQKGGSTTE   174
126-14                VVTLKYDKATNWSKTDTYSLSEKVTTKNKFQWPLVGETELAIEIAASQSWASQKGGSTTE   298
312M                  VVTLKYDKATNWSKTDTYSLSEKVTTKNKFQWPLVGETELAIEIAASQSWASQKGGSTTE   298
VBF557                VVTLKYDKATNWSKTDTYSLSEKVTTKNKFQWPLVGETELAIEIAASQSWASQKGGSTTE   298
ERR1305902-bin.15     VVTLKYDKATNWSKTDTYSLSEKVTTKNKFQWPLVGETELAIEIAASQSWASQKGGSTTE   298
                      ********:*:*********.* *  *..** * *..** ::**:::****.*:* .* *


A20                   TVSVEARPTVPPHSSLPVRVALYKSNISYPYEFKAEVNYDLTMKGFLRWGGNAWYTHPDN   357
A20-10                TVSVEARPTVPPHSSLPVRVALYKSNISYPYEFKAEVNYDLTMKGFLRWGGNAWYTHPEN   358
A20-12                TVSVEARPTVPPHSSLPVRVALYKSNISYPYEFKAEVNYDLTMKGFLRWGGNAWYTHPDN   358
A20-14                TVSVEARPTVPPHSSLPVRVALYKSNISYPYEFKAEVNYDLTMKGFLRWGGNAWYTHPEN   358
A20-17                TVSVEARPTVPPHSSLPVRVALYKSNISYPYEFKAEVNYDLTMKGFLRWGGNAWYTHPEN   358
A20-5                 TVSVEARPTVPPHSSLPVRVALYKSNISYPYEFKAEVNYDLTMKGFLRWGGNAWYTHPEN   358
A20-8                 TVSVEARPTVPPHSSLPVRVALYKSNISYPYEFKAEVNYDLTMKGFLRWGGNAWYTHPEN   358
A21                   TVSVEARPTVPPHSSLPVRVALYKSNISYPYEFKAEVNYDLTMKGFLRWGGNAWYTHPEN   358
A21-10                TVSVEARPTVPPHSSLPVRVALYKSNISYPYEFKAEVNYDLTMKGFLRWGGNAWYTHPEN   358
A21-11                TVSVEARPTVPPHSSLPVRVALYKSNISYPYEFKAEVNYDLTMKGFLRWGGNAWYTHPEN   358
A21-13                TVSVEARPTVPPHSSLPVRVALYKSNISYPYEFKAEVNYDLTMKGFLRWGGNAWYTHPEN   358
A21-14                TVLVEARPTVPPYSSLPVRIALYKSNISYPYEFKAEVNYDLTMKGFLRRNGNAWYTHPEN   359
A21-15                TVSVEARPTVPPHSSLPVRVALYKSNISYPYEFKAEVNYDLTMKGFLRWGGNAWYTHPEN   358
A21-16                TVSVEARPTVPPHSSLPVRVALYKSNISYPYEFKAEVNYDLTMKGFLRWGGNAWYTHPEN   358
A21-19                TVSVEARPTVPPHSSVPVRVALYKSNISYPYEFKAEVNYDLTMKGFLRWGGNAWYTHPAN   358
A21-4                 TVSVEARPTVPPHSSLPVRVALYKSNISYPYEFKAEVNYDLTMKGFLRWGGNAWYTHPDN   358
A21-5                 TVSVEARPTVPPHSSLPVRVALYKSNISYPYEFKAEVNYDLTMKGFLRWGGNAWYTHPEN   358
A21-6                 TVSVEARPTVPPHSSLPVRVALYKSNISYPYEFKAEVNYDLTMKGFLRWGGNAWYTHPTN   358
A21-8                 TVSVEARPTVPPHSSLPVRVALYKSNISYPYEFKAEVNYDLTMKGFLRWGGNAWYTHPEN   358
A26                   TVSVEARPTVPPHSSLPVRVALYKSNISYPYEFKAEVNYDLTMKGFLRWGGNAWYTHPDN   358
A27                   TVSVEARPTVPPHSSLPVRVALYKSNISYPYEFKAEVNYDLTMKGFLRWGGNAWYTHPEN   358
A29V                  TVSVEARPTVPPHSSLPVRVALYKSNISYPYEFKAEVNYDLTMKGFLRWGGNAWYTHPEN   358
A7                    TVSVEARPTVPPHSSLPVRVALYKSNISYPYEFKAEVNYDLTMKGFLRWGGNAWYTHPEN   358
A8                    TVSVEARPTVPPHSSLPVRVALYKSNISYPYEFKAEVNYDLTMKGFLRWGGNAWYTHPEN   358
A9                    TVSVEARPTVPPHSSLPVRVALYKSNISYPYEFKAEVNYDLTMKGFLRWGGNAWYTHPDN   358
BC88                  TVSVEARPTVPPHSSLPVRVALYKSNISYPYEFKAEVNYDLTMKGFLRWGGNAWYTHPEN   357
FC951                 TVSVEARPTVPPHSSLPVRVALYKSNISYPYEFKAEVNYDLTMKGFLRWGGNAWYTHPEN   234
126-14                TVSVEARPTVPPHSSLPVRVALYKSNISYPYEFKAEVNYDLTMKGFLRWGGNAWYTHPEN   358
312M                  TVSVEARPTVPPHSSLPVRVALYKSNISYPYEFKAEVNYDLTMKGFLRWGGNAWYTHPEN   358
VBF557                TVSVEARPTVPPHSSLPVRVALYKSNISYPYEFKAEVNYDLTMKGFLRWGGNAWYTHPEN   358
ERR1305902-bin.15     TVSVEARPTVPPHSSLPVRVALYKSNISYPYEFKAEVNYDLTMKGFLRWGGNAWYTHPEN   358
                      ** *********:**:***:**************************** .******** *
```

```
A20                    RPTREHTFAVGPFRDKASSIRYQWDKRYIPGEVKWWDWNWTISEYGLSTMQNNLGRVLRP   417
A20-10                 RPTWEHTFAVGPFRDKASSIRYQWDKRYIPGEVKWWDWNWTISEYGLSTMQNNLGRVLRP   418
A20-12                 RPTWEHTFAVGPFRDKASSIRYQWDKRYIPGEVKWWDWNWTISEYGLSTMQNNLGRVLRP   418
A20-14                 RPTWEHTFAVGPFRDKASSIRYQWDKRYIPGEVKWWDWNWTISEYGLSTMQNNLGRVLRP   418
A20-17                 RPTWEHTFAVGPFRDKASSIRYQWDKRYIPGEVKWWDWNWTISEYGLSTMQNNLGRVLRP   418
A20-5                  RPTWEHTFAVGPFRDKASSIRYQWDKRYIPGEVKWWDWNWTISEYGLSTMQNNLGRVLRP   418
A20-8                  RPTWEHTFAVGPFRDKASSIRYQWDKRYIPGEVKWWDWNWTISEYGLSTMQNNLGRVLRP   418
A21                    RPTWEHTFAVGPFRDKASSIRYQWDKRYIPGEVKWWDWNWTISEYGLSTMQSNLGRVLRP   418
A21-10                 RPTWEHTFAVGPFRDKASSIRYQWDKRYIPGEVKWWDWNWTISEYGLSTMQNNLGRVLRP   418
A21-11                 RPTWEHTFAVGPFRDKASSIRYQWDKRYIPGEVKWWDWNWTISEYGLSTMQNNLGRVLRP   418
A21-13                 RPTWEHTFAVGPFRDKASSIRYQWDKRYIPGEVKWWDWNWTISEYGLSTMQNNLGRVLRP   418
A21-14                 GPTLEHTFAVGPFRDKASSIRYQWDKRYIPGEMKWWDWNWAISEYGLSTMQSNLGLVLRT   419
A21-15                 RPTWEHTFAVGPFRDKASSIRYQWDKRYIPGEVKWWDWNWTISEYGLSTMQNNLGRVLRP   418
A21-16                 RPTWEHTFAVGPFRDKASSIRYQWDKRYIPGEVKWWDWNWTISEYGLSTMQNNLGRVLRP   418
A21-19                 RPTWEHTFAIGPFRDKASSIRYQWDKRYIPGEVKWWDWNWTISEYGLSTMQSNLGRVLRP   418
A21-4                  RPTWEHTFAVGPFRDKASSIRYQWDKRYIPGEVKWWDWNWTISEYGLSTMQNNLGRVLRP   418
A21-5                  RPTWEHAFAVGPFRDKASSIRYQWDKRYIPGEVKWWDWNWTISEYGLSTMQNNLGRVLRP   418
A21-6                  RPTWEHTFAVGPFRDKASSIRYQWDKRYIPGEVKWWDWNWTISEYGLSTMQNNLGRVLRP   418
A21-8                  RPTWEHTFAVGPFRDKASSIRYQWDKRYIPGEVKWWDWNWTISEYGLSTMQNNLGRVLRP   418
A26                    RPTWEHTFAVGPFRDKASSIRYQWDKRYIPGEVKWWDWNWTISEYGLSTMQNNLGRVLRP   418
A27                    RPTWEHTFAVGPFRDKASSIRYQWDKRYIPGEVKWWDWNWTISEYGLSTMQNNLGRVLRP   418
A29V                   RPTWEHTFAVGPFRDKASSIRYQWDKRYIPGEVKWWDWNWTISEYGLSTMQNNLGRVLRP   418
A7                     RPTWEHTFAVGPFRDKASSIRYQWDKRYIPGEVKWWDWNWTISEYGLSTMQNNLGRVLRP   418
A8                     RPTWEHTFAVGPFRDKASSIRYQWDKRYIPGEVKWWDWNWTISEYGLSTMQNNLGRVLRP   418
A9                     RPTWEHTFAVGPFRDKASSIRYQWDKRYIPGEVKWWDWNWTISEYGLSTMQNNLGRVLRP   418
BC88                   RPTWEHTFAVGPFRDKASSIRYQWDKRYIPGEVKWWDWNWTISEYGLSTMQNNLGRVLRP   417
FC951                  RPTWEHTFAVGPFRDKASSIRYQWDKRYIPGEVKWWDWNWTISEYGLSTMQNNLGRVLRP   294
126-14                 RPTWEHTFAVGPFRDKASSIRYQWDKRYIPGEVKWWDWNWTISEYGLSTMQNNLGRVLRP   418
312M                   RPTWEHTFAVGPFRDKASSIRYQWDKRYIPGEVKWWDWNWTISEYGLSTMQNNLGRVLRP   418
VBF557                 RPTWEHTFAVGPFRDKASSIRYQWDKRYIPGEVKWWDWNWTISEYGLSTMQNNLGRVLRP   418
ERR1305902-bin.15      RPTWEHTFAVGPFRDKASSIRYQWDKRYIPGEVKWWDWNWTISEYGLSTMQNNLGRVLRP   418
                        ** **:**:**********************:*******:**********.*** ***
```

```
A20                    IRSAVTGDFYAESQFAGDIEIGQPQTRSAASAQLRNASAEGVALTGVDLDQEALANEGFG   477
A20-10                 IRSAVTGDFYAESQFAGDIEIGQPQTRSAKAAQLRSASADEVALTGVDLDSEALANEGFG   478
A20-12                 IRSAVTGDFYAESQFAGDIEIGQPQTRSAASAQLRHASAEGVALTGVDLDQEALANEGFG   478
A20-14                 IRSAVTGDFYAESQFAGDIEIGQPQTRSAKAAQLRSASADEVALTGVDLDSEALANEGFG   478
A20-17                 IRSAVTGDFYAESQFAGDIEIGQPQTRSAKAAQLRNSSAEGVALTGVDLDSEALASEGFG   478
A20-5                  IRSAVTGDFYAESQFAGDIEIGQPQTRSAKSAQLRSASADEVALTGVDLDSEALANEGFG   478
A20-8                  IRSAVTGDFYAESQFAGDIEIGQPQTRSAKSAQLRSASADEVALTGVDLDSEALANEGFG   478
A21                    IRSAVTGDFYAESQFAGDIEIGQPQTRSAASAQLRNASAEGVALTGVDLDSEALANEGFG   478
A21-10                 IRSAVTGDFYAESQFAGDIEIGQPQTRSAKSAQLRSASADEVALTGVDLDSEALANEGFG   478
A21-11                 IRSAVTGDFYAESQFAGDIEIGQPQTRSAASAQLRNASAEGVALTGVDLDSEALANEGFG   478
A21-13                 IRSAVTGDFYAESQFAGDIEIGQPQTRSAKAAQLRSASAEGVALTGVDLDQEALANEGFG   478
A21-14                 VHASVTGDFYAESQFAGDIEIGLPPTTSTKA-LLRSASVNMVELTDLKLDRKNLASEGFG   478
A21-15                 IRSAVTGDFYAESQFAGDIEIGQPQTRSAKAAQLRSASADEVALTGVDLDSEALANEGFG   478
A21-16                 IRSAVTGDFYAESQFAGDIEIGQPQTRSAKAAQLRSASADEVALTGVDLDSEALANEGFG   478
A21-19                 IRSAVTGDFYAESQFAGDIEIGQPQSGSTKAAQLRSASADEVALTGVDLDSEALANEGFG   478
A21-4                  IRSAVTGDFYAESQFAGDIEIGQPQTRSAKAAQLRSASADEVALTGVDLDSEALANEGFG   478
A21-5                  IRSAVTGDFYAESQFAGDIEIGQPQTRSAKAAQLRSASADEVALTGVDLDSEALANEGFG   478
A21-6                  IRSAVTGDFYAESQFAGDIEIGQPQTRSAKAAQLRNSSAEGVALTGVDLDSETLASEGFG   478
A21-8                  IRSAVTGDFYAESQFAGDIEIGQPQTRSAASAQLRNASAEGVALTGVDLDQEALANEGFG   478
A26                    IRSAVTGDFYAESQFAGDIEIGQPQTRSAKAAQLRSASADEVALTGVDLDSEALANEGFG   478
A27                    IRSAVTGDFYAESQFAGDIEIGQPQTRSAASAQLRNASAEGVALTGVDLDQEALANEGFG   478
A29V                   IRSAVTGDFYAESQFAGDIEIGQPQTRSAASAQLRNASAEGVALTGVDLDQEALANEGFG   478
A7                     IRSAVTGDFYAESQFAGDIEIGQPQTRSAKAAQLRSASADEVALTGVDLDSEALANEGFG   478
A8                     IRSAVTGDFYAESQFAGDIEIGQPQTRSAKAAQLRSASADEVALTGVDLDSEALANEGFG   478
A9                     IRSAVTGDFYAESQFAGDIEIGQPQTRSAKAAQLRSASADEVALTGVDLDSEALANEGFG   478
BC88                   IRSAVTGDFYAESQFAGDIEIGQPQTRSAKAAQLRSASADEVALTGVDLDSEALANEGFG   477
FC951                  IRSAVTGDFYAESQFAGDIEIGQPQTRSAKAAQLRSASADEVALTGVDLDSEALANEGFG   354
126-14                 IRSAVTGDFYAESQFAGDIEIGQPQTRSAKAAQLRSASADEVALTGVDLDSEALANEGFG   478
312M                   IRSAVTGDFYAESQFAGDIEIGQPQTRSAKAAQLRSASADEVALTGVDLDSEALANEGFG   478
VBF557                 IRSAVTGDFYAESQFAGDIEIGQPQTRSAASAQLRNASAEGVALTGVDLDQEALANEGFG   478
ERR1305902-bin.15      IRSAVTGDFYAESQFAGDIEIGQPQTRSAKAAQLRSASADEVALTGVDLDSEALANEGVG   478
                       ::::****************** * : *: :  ** :*.: * **.:.** : **.**.*
```

```
A20                    NVSLTVTPAY     487
A20-10                 NVSLTIVPVQ     488
A20-12                 NVSLTVTPAY     488
A20-14                 NVSLTIVPVQ     488
A20-17                 NVSLTIAPVQ     488
A20-5                  NVSLTIVPVQ     488
A20-8                  NVSLTIVPVQ     488
A21                    NVSLTIVPVQ     488
A21-10                 NVSLTIVPVQ     488
A21-11                 NVSLTIVPVQ     488
A21-13                 NVSLTIVPVQ     488
A21-14                 NVSLTLAPAH     488
A21-15                 NVSLTIVPVQ     488
A21-16                 NVSLTIVPVQ     488
A21-19                 NVSLTIVPVQ     488
A21-4                  NVSLTIVPVQ     488
A21-5                  NVSLTIVPVQ     488
A21-6                  NVSLTMAPVQ     488
A21-8                  NVSLTIVPVQ     488
A26                    NVSLTIVPVQ     488
A27                    NVSLTIVPVQ     488
A29V                   NVSLTVTPAY     488
A7                     NVSLTIVPVQ     488
A8                     NVSLTIVPVQ     488
A9                     NVSLTIVPVQ     488
BC88                   NVSLTIVPVQ     487
FC951                  NVSLTIVPVQ     364
126-14                 NVSLTIVPVQ     488
312M                   NVSLTIVPVQ     488
VBF557                 NVSLTIVPVQ     488
ERR1305902-bin.15      NVSLTIVPVQ     488
                       *****:.*.
```

Additional file 2 Protein sequence alignment of aerolysin from *Aeromonas veronii* strains isolated from patients with gastrointestinal disease. Protein alignment was performed with Clustal Omega.
